# Genetic dissection of stem WSC accumulation and remobilization in wheat (*Triticum aestivum* L.) under terminal drought stress

**DOI:** 10.1186/s12863-020-00855-1

**Published:** 2020-04-29

**Authors:** Mengfei Li, Yuan Liu, Jingfu Ma, Peipei Zhang, Caixiang Wang, Junji Su, Delong Yang

**Affiliations:** 1 Gansu Provincial Key Lab of Aridland Crop Science, Lanzhou, 730070 Gansu China; 2 College of Life Science and Technology, Gansu Agricultural University, Lanzhou, 730070 Gansu China

**Keywords:** *Triticum aestivum*, Stem water-soluble carbohydrates, QTL mapping, Genetic dissection, Terminal drought stress

## Abstract

**Background:**

The accumulation and remobilization of stem water soluble carbohydrates (WSC) are determinant physiological traits highly influencing yield potential in wheat against drought stress. However, knowledge gains of the genetic control are still limited. A hexaploid wheat population of 120 recombinant inbred lines were developed to identify quantitative trait loci (QTLs) and to dissect the genetic basis underlying eight traits related to stem WSC under drought stress (DS) and well-watered (WW) conditions across three environments.

**Results:**

Analysis of variance (ANOVA) revealed larger environmental and genotypic effects on stem WSC-related traits, indicating moderate heritabilities of 0.51–0.72. A total of 95 additive and 88 pairs of epistatic QTLs were identified with significant additive and epistatic effects, as well as QTL× water environmental interaction (QEI) effects. Most of additive QTLs and additive QEIs associated with drought-stressed environments functioned genetic effects promoting pre-anthesis WSC levels and stem WSC remobilization to developing grains. Compared to other genetic components, both genetic effects were performed exclusive contributions to phenotypic variations in stem WSC-related traits. Nineteen QTL clusters were identified on chromosomes 1B, 2A, 2B, 2D, 3B, 4B, 5A, 6A, 6B and 7A, suggestive of the genetic linkage or pleiotropy. Thirteen additive QTLs were detectable repeatedly across two of the three water environments, indicating features of stable expressions. Some loci were consistent with those reported early and were further discussed.

**Conclusion:**

Stem WSC-related traits were inherited predominantly by additive and QEI effects with a moderate heritability. QTL cluster regions were suggestive of tight linkage or pleiotropy in the inheritance of these traits. Some stable and common loci, as well as closely linked molecular markers, had great potential in marker-assisted selection to improve stem WSC-related traits in wheat, especially under drought-stressed environments.

## Background

Wheat (*Triticum aestivum* L.), one of the most important cereal crops, is widely grown in semiarid and arid areas around the world. As increasing the precipitation variability with frequent episodes of drought in recent decades, wheat crops often suffer from erratic water deficit during the growing season [1]. In particular, the terminal drought scenarios, occurring during reproductive and grain-filling phases, are the most detrimental to photosynthetic performance [2] and grain set and development [3], which resulted in a substantial reduction in final grain yield [4, 5]. Therefore, improving water-use efficiency (WUE) by incorporating drought-tolerant traits into elite breeding germplasm is an important objective for the genetic improvement in wheat in water-deficit environments [6].

An alternative to screening for grain yield and its stability under different water-stressed environments is to identify useful physiological adaptations to improve WUE in wheat and other cereal crops [7]. Of these, water soluble carbohydrates (WSC), composed mainly of fructan together with minor components of sucrose and hexose [8, 9], have been considered a promising physiological trait indicative of drought tolerance in wheat crops [10–12]. This attributes to WSC acting as osmolytes to enhance water retention [13] and protect plants from drought stress by scavenging reactive oxygenspecies [14] and by stabilizing cellular membranes [15]. As the dominant carbon source, WSC reserves can contribute to grain development and improve grain yield potential, when active photosynthesis is inhibited by terminal drought [10, 16, 17]. Previous studies have reported that WSC reserves can continuously accumulate before anthesis and peak at 7–20 days after anthesis in wheat crops [18, 19], when photosynthetic tissues synthesize WSC at a rate greater than the requirement for various sinks [10, 18]. The excess WSC is stored mainly in stems and leaf sheaths [18], usually accounting for more than 40% of total dry weight of stems [8, 19]. Its remobilization, afterwards, is available for use in plant growth or respiration [20], but more for contribution to final grain yield [8, 17]. Under the terminal drought condition, stem WSC remobilization during the grain-filling phase can contribute as much as 30–50% of grain yield [8, 11, 17], and even potentially up to 70% in some elite cultivars [21, 22].

There are considerable genotypic variations in stem WSC accumulation and remobilization observed in wheat under individual water environments [8, 11, 19, 23]. Besides, genotypic ranking among diverse wheat cultivars for stem WSC content is consistent across a range of environments, indicating moderate to high broad-sense heritabilities (*h*_B_^2^) of 0.46–0.93 [22–26]. This indicates that stem WSC levels are genetically determined and that selection for high-level WSC should be possible at the early generation stage of a breeding program [19, 22, 26, 27]. However, some studies also show wide fluctuations in these traits underlined by strong genotype × environment interaction (GEI), suggesting stem WSC reserves were highly sensitive to water environments [6, 28, 29]. Consequently, further knowledge gains of genotypic variations, genomic locations, and molecular genetic basis in stem WSC are critical for well-understanding yield-limiting factors and for improving yield potential in wheat, especially in water-deficit environments [16, 22, 29]. During the last decades, a wealth of quantitative trait loci (QTLs) for stem WSC-related traits have been identified in diverse genetic populations and field-growing environments by the strategy of linkage genetic analysis [22, 28, 30–34] and genome-wide association [6, 26, 29, 35, 36]. These studies have reported that QTLs for stem WSC accumulation and remobilization could have different expression patterns in response to different growth stages or environments. Most of additive QTLs significantly interacted with water environments, suggesting that stable molecular markers for these traits are essential to understand its genetic basis. Furthermore, Rebetzke et al. [22] identified fewer significant QTLs for stem WSC levels in three doubled-haploid (DH) populations, while sizes of individual genetic effects varied between populations but were repeatable across environments. Several genomic regions were common across populations including those associated with plant height or anthesis date. Some important chromosomal regions governing stem WSC-related traits were also found to overlap with locations of QTLs for yield-related traits and drought tolerance [28, 32–34], suggesting pleiotropic effects in the inheritance of these traits.

Although stem WSC-related traits proved to be quantitative, knowledge gains of the molecular genetic basis are still limited. In the current study, a hexaploid wheat population of 120 recombinant inbred lines (RIL) was employed to map QTLs for eight traits related to stem WSC accumulation and remobilization across six water environments. The objectives were to identify putative QTLs for stem WSC-related traits, and to estimate the genetic control involving in interaction effects with water environments. The findings will provide a better understanding of the polygene-inherited mechanisms governing stem WSC-related traits in wheat under water-deficit environments, and should benefit genetic improvement of drought tolerance in wheat by pyramiding favorable QTLs.

## Results

### Statistical analysis of phenotypic assessment

The means of eight stem WSC-related traits averaged across three experimental sites were employed to evaluate phenotypic variations for the RIL population and parents in response to drought-stressed (DS) and well-watered (WW) conditions, respectively. As summarized in Table 1, both parents differed significantly from tested traits (*P* < 0.05). Most of them evaluated in Longjian 19, except for stem WSC concentration at the maturity stage (WSCm), were much higher than those in Q9086. Across all water treatments, mean values of the RIL population were intermediate between the parents and showed wide phenotypic variability. The corresponding coefficients of variation ranged from 13.93 to 51.37% in the DS and from 11.87 to 58.68% in the WW. Some progenies had extreme values beyond either parent. Both skewness and kurtosis values were less than 1.0 observed in almost all treatments, suggesting the continuous variation and transgressive segregation for tested traits in the RIL population.

**Table 1:** Summary statistics of stem WSC-related traits in the parents and the wheat RIL population under drought-stressed (DS) and well-watered (WW) conditions across different environments

Trait	Water regime	Parents		RILs						
		Longjian19	Q9086	Mean	Min.	Max.	CV (%)	Skew.	Kurt.	*h*^2^_B_
WSCf (mg.g^− 1^ DW)	DS	127.33	113.80^*^	115.34^*^	51.39	175.32	21.11^*^	−0.25	− 0.17	0.54
	WW	102.49	87.06^*^	98.42	42.43	172.17	18.86	0.13	0.36	0.63
WSCg (mg.g^−1^ DW)	DS	185.38	157.84^*^	168.73^*^	71.36	257.92	17.78	−0.36	0.28	0.69
	WW	151.06	110.59^**^	148.55	81.82	254.85	16.81	0.35	0.73	0.72
WSCm (mg.g^−1^ DW)	DS	34.17	43.82^**^	40.53^**^	7.32	95.90	33.26^**^	0.79	0.88	0.70
	WW	73.56	104.53^**^	80.65	19.81	148.94	24.13	0.32	0.02	0.55
WRRpr (%)	DS	69.29	54.46^**^	64.78^**^	32.63	90.36	13.93^**^	−0.60	0.28	0.57
	WW	19.04	13.39^**^	18.47	0.92	75.41	48.46	0.70	1.50	0.59
WRRps (%)	DS	31.38	24.52^**^	25.00^**^	3.65	71.75	38.78^**^	0.33	0.11	0.51
	WW	44.75	31.71^**^	42.68	1.92	73.53	30.61	0.15	−0.22	0.64
WCRpr (%)	DS	16.40	9.54^**^	15.11^**^	3.65	27.92	35.92^**^	0.29	−0.67	0.60
	WW	6.24	2.38^**^	3.48	0.18	14.78	58.68	0.98	1.40	0.61
WCRps (%)	DS	8.42	4.51^**^	5.70^**^	0.75	27.98	51.37^**^	0.57	−0.23	0.54
	WW	17.73	8.74^**^	8.93	0.21	26.25	29.27	0.58	−0.49	0.61
GWMS (g)	DS	1.05	0.89^*^	0.98^**^	0.48	1.52	18.92^**^	0.05	−0.40	0.73
	WW	1.43	1.18^*^	1.31	0.83	1.71	11.87	0.12	−0.27	0.65

The phenotypic data for each trait is the mean averaged across three experimental sites under drought-stressed (DS) and well-watered (WW) conditions at Anning farm station, Gansu, China (103°51′E, 36°04′N, 1600 m ASL) in 2012–2013 (E1), at Yongdeng farm station, Gansu, China (103°18′E, 36°42′N, 1950 m ASL) in 2013–2014 (E2), and at Yuzhong farm station, Gansu, China (104°07′E, 35°51′N, 1900 m ASL) in 2014–2015 (E3). The asterisks in the column of “parent Q9086” represent significant differences in phenotypic data between two parents by the *F* test. The asterisks in the columns of “mean” and “CV (%)” represent significant differences in phenotypic data between two water conditions by the *F* test. ^*^P < 0.05, ^**^P < 0.01. *WSC* water-soluble carbohydrate concentration, *WSCf* WSC at the anthesis stage, *WSCg* WSC at the grain-filling stage, *WSCg* WSC at the grain-filling stage, *WSCm* WSC at the maturity stage, *WRRpr* pre-anthesis WSC remobilization rate, *WRRps* post-anthesis WSC remobilization rate, *WCRpr* pre-anthesis WSC contribution rate, *WCRps* post-anthesis WSC contribution rate, *GWMS* grain weight of main spike, *Min* minimum, *Max* maximum, *CV* coefficient of variation, *Skew* skewness, *Kurt* kurtosis, *h*^*2*^_*B*_ broad-sense heritability

The analysis of variance (ANOVA) showed that phenotypic variances for stem WSC-related traits in the RIL population and two parents reached at the significant levels (*P* < 0.05), with the exception of individual two- and three-way interactions (Table S1 and S2). In comparison, water condition had larger effects on all tested traits, followed by genotype and site, whereas, for other factors, the effects were relatively smaller and even not significant somewhat (*P* > 0.05). However, stem WSC-related traits differed significantly from two water conditions (Table 1). Most of the traits for all genotypes had higher phenotypic means (except for WSCm and grain weight of main spike (GWMS)), and greater coefficients of variation (except for stem WSC remobilization rates at pre-anthesis (WRRpr) and WSC contribution rates to GWMS at pre-anthesis (WCRpr)) under the DS than those under the WW. Both WRRpr and WCRpr under the DS were consistently higher than stem WSC remobilization rates at post-anthesis (WRRps) and WSC contribution rates to GWMS at post-anthesis (WCRps), respectively, whereas it was reversed to those under the WW. The *h*^2^_B_ for most of traits (except for WSCm and GWMS) under the DS was slightly lower than those under the WW, varying from 0.51 to 0.70 in the WW and from 0.55 to 0.72 in the DS. The results indicated that water environment strongly influenced stem WSC-related traits, which further revealed the genetic nature of their complex quantitative traits.

### Phenotypic correlation analysis

**Fig. 1:**
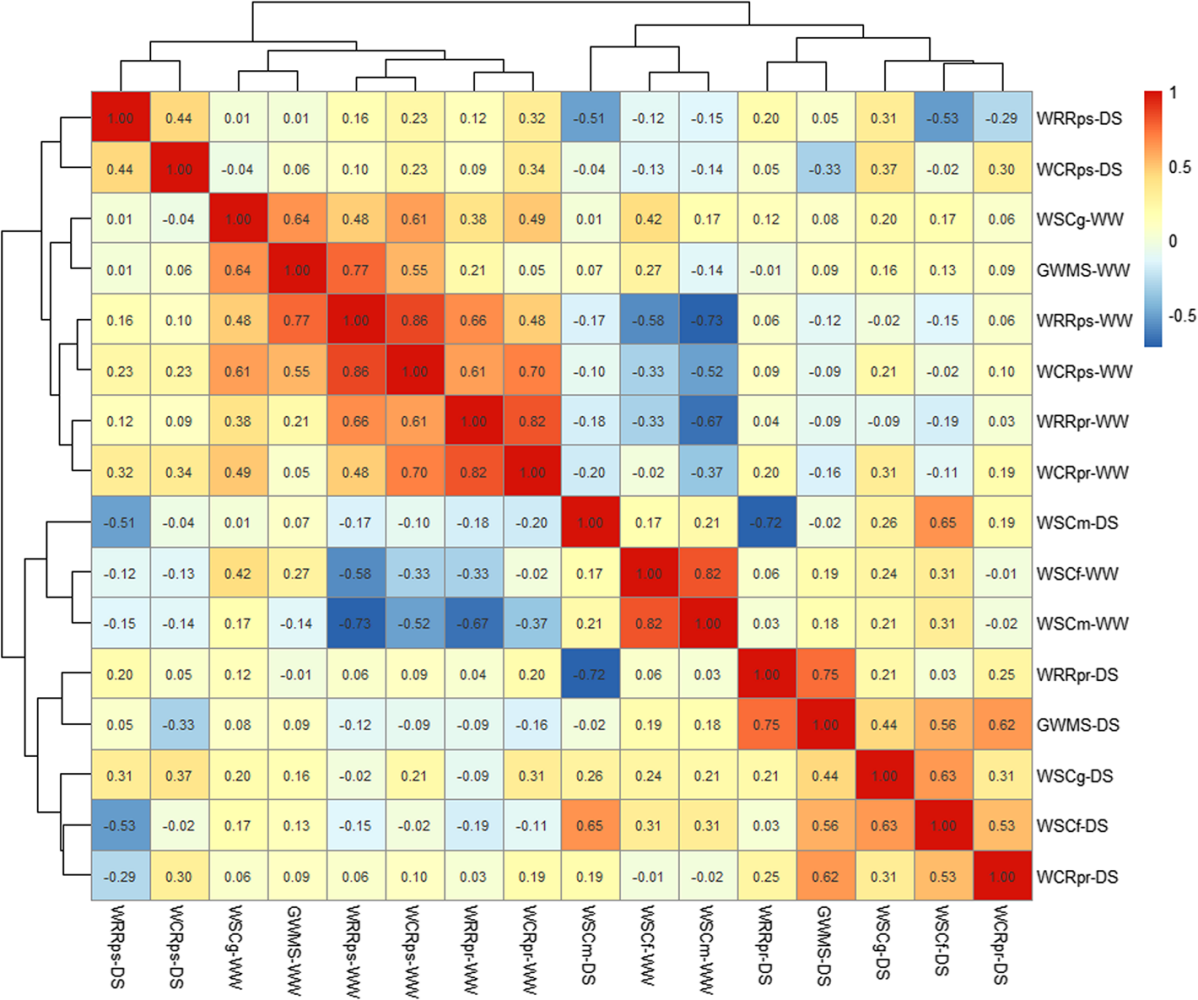
Heatmap summarizing Pearson correlation between stem WSC-related traits in the wheat RIL population under drought-stressed (DS) and well-watered (WW) conditions across different environments. The stem WSC-related traits evaluated herein involved in grain weight of main spike (GWMS); water-soluble carbohydrate (WSC) concentration at the anthesis stage (WSCf), at the grain-filling stage (WSCg) and at the maturity stage (WSCm); WSC remobilization rate at the pre-anthesis (WRRpr) and at the post-anthesis (WRRps); WSC remobilization rate at the pre-anthesis (WCRpr) and at the post-anthesis (WCRps)

Phenotypic correlation coefficients (*r*) among tested traits for the RIL population under DS and WW conditions were shown in Fig. 1. Most of traits were positively correlated with each other in each water condition, and some correlations reached at significant levels (*P* < 0.05). Nevertheless, correlations among each trait differed greatly under the DS from that under the WW. Under the DS, there were positive and significant correlations identified among stem WSC concentration at the anthesis stage (WSCf) and at the grain filling stage (WSCg), WCRpr and GWMS (*r =* 0.31 to 0.63). Besides, the positive correlations were also significant between GWMS and WRRpr (*r* = 0.75), between WSCf and WSCm (*r* = 0.65), and WRRps and WCRps (*r* = 0.44), respectively. Under the WW, positive and significant correlations were found among WSCg, WRRpr, WRRps, WCRpr, WCRps and GWMS (*r* = 0.38 to 0.86), except for weak correlations of GWMS with WRRpr and WCRpr. Similar to the DS, WSCf was positively correlated with WSCm (*r* = 0.82). In addition, a few of traits were negatively and significantly correlated with each other between DS and WW conditions. This situation, under the DS, occurred between WRRps and WSCf (*r* = − 0.53) and WSCm (*r* = − 0.51), between WRRpr and WSCm (*r* = − 0.72), and between WCRps and GWMS (*r* = − 0.33), respectively. Under the WW, they involved in WSCm with WRRpr, WRRps, WCRpr and WCRps (*r* = − 0.37 to − 0.73), and WSCf with WRRpr, WRRps and WCRps (*r* = − 0.33 to − 0.58), respectively. By comparison, phenotypic correlations for most traits between DS and WW conditions were weak and variable, suggesting that stem WSC accumulation and remobilization were highly influenced by the water environment.

### QTLs mapping and QTL × water environment interactions

**Fig. 2:**
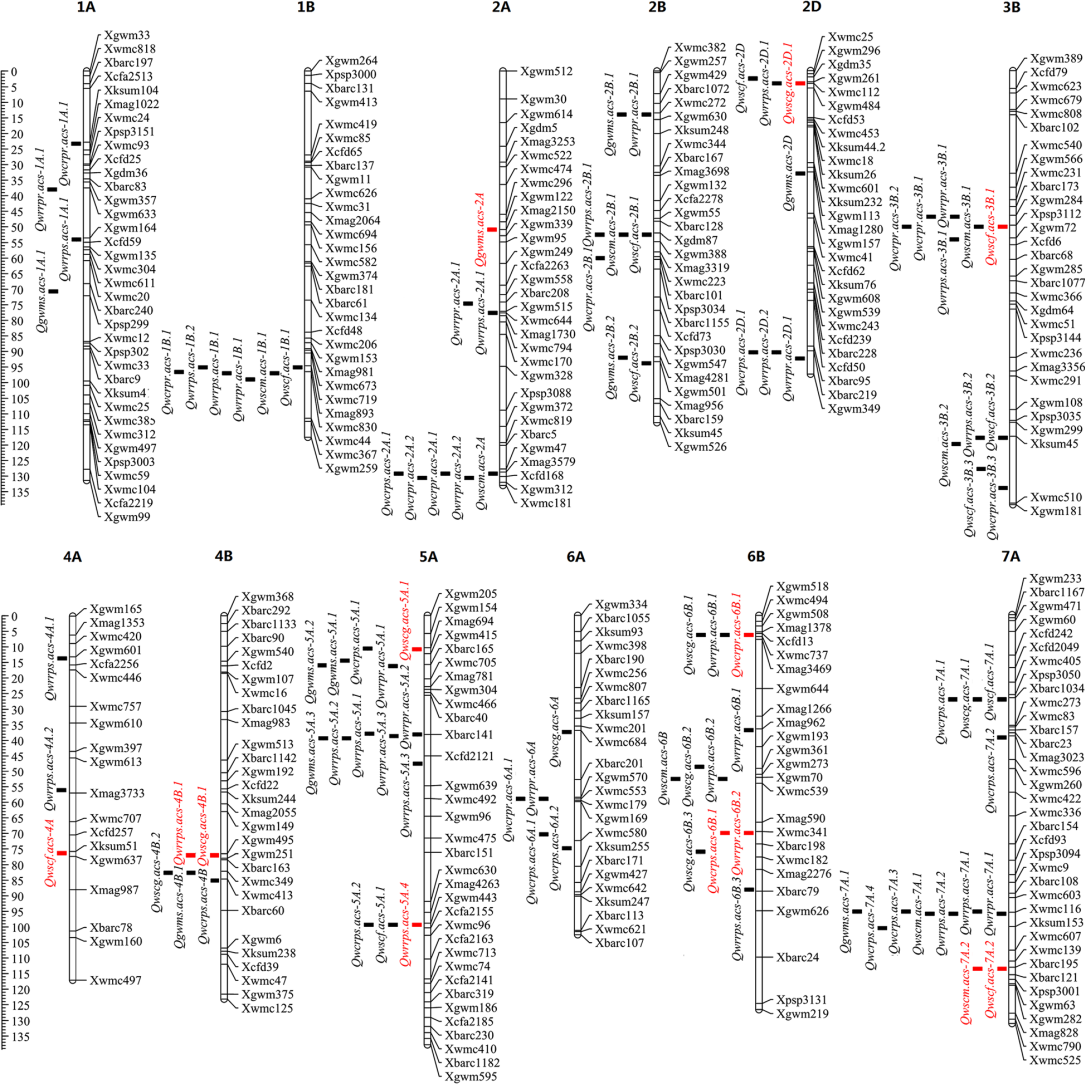
Additive QTLs and QTLs cluster for stem WSC-related traits in the wheat RIL population. The black squares are QTLs expressed only in one environment and red squares are QTLs expressed repeatedly in two environments for stem WSC-related traits

Considering the eight traits evaluated in DS and WW conditions across environments E1 to E3, a total of 95 additive QTLs were identified and widely mapped on chromosomes 1A, 1B, 2A, 2B, 2D, 3B, 4A, 4B, 5A, 6A, 6B and 7A (Table S3, Fig. 2). These loci individually accounted for 0.83 to 23.14% of phenotypic variance. The number of QTLs detected for each trait ranged from 8 (WSCm) to 21 (WRRps). Of these, 50 (52.6%) had positive additive (*a*) effects, indicating favorable allele contribution from the parent Longjian19. In contrast, the other 45 (47.4%) showed negative *a* effects with favorable alleles from Q9086. For each trait, more favorable alleles (60.0–85.7%) for WSCf, WSCg, WRRpr and WCRpr were derived from Longjian19 and those for WRRps (66.7%) and GWMS (90.0%) were from Q9086. This indicated that favorable alleles governing stem WSC-related traits were almost unevenly contributed by the parents. In addition, 82 QTLs (86.3%) were identified in single environments, and only 13 QTLs (13.7%) were detectable repeatedly across two of the three water environments, suggestive of the features of stable expressions. Both WSCf and WSCg showed the highest number with three stable QTLs, followed by two for WRRps and one for each of the other five traits (Table 2). With the exception of 22 loci (23.2%) expressed only significant *a* effects, the other 73 (76.8%) showed QTL× water environmental interaction (QEI) with drought stress, indicating their significant additive QEI (*ae*) effects. Among them, 43 loci, especially for WSCf, WSCg, WRRpr and WCRpr, contributed positive *ae* effects, whereas, for the other 30 loci mainly for WSCm, *ae* effects were negative. The additive QEIs in both groups individually accounted for phenotypic variance from 0.99 to 18.09% and from 0.81 to 19.86%, respectively.

**Table 2:** Stable additive QTLs for stem WSC-related traits across water environments in the wheat RIL population

Trait	QTL	Flanking markers	Site (cM)	Environ.	*a*	*R*^2^(*a*)%
WSCf	*Qwscf.acs-3B.1*	Xpsp3112-Xgwm72	49.6	**E1**/E3	−3.55^***^/− 3.48^***^	6.96/8.09
	*Qwscf.acs-4A*	Xksum51-Xgwm637	75.7	**E1**/E3	4.07^***^/3.77^***^	9.14/9.58
	*Qwscf.acs-7A.2*	Xbarc195-Xbarc121	113.2	E2/E3	1.59^***^/2.12^***^	3.42/4.12
WSCg	*Qwscg.acs-2D.1*	Xgwm261-Xwmc112	3.5	**E1**/E2	−2.45^***^/−3.45^***^	3.92/4.62
	*Qwscg.acs-4B.1*	Xgwm495-Xgwm251	76.5	E2/**E3**	−3.55^***^/−2.36^***^	4.91/3.08
	*Qwscg.acs-5A.1*	Xmag694-Xgwm415	10.4	**E2**/**E3**	2.26^***^/3.32^***^	2.97/3.83
WSCm	*Qwscm.acs-7A.2*	Xbarc195-Xbarc121	113.2	E1/**E3**	2.29^***^/2.80^***^	3.48/8.94
WRRpr	*Qwrrpr.acs-6B.2*	Xwmc341-Xbarc198	69.4	E1/**E3**	1.68^**^/1.96^***^	5.49/6.66
WRRps	*Qwrrps.acs-4B.1*	Xgwm495-Xgwm251	76.5	**E1**/**E2**	−1.50^***^/−3.05^***^	1.75/5.31
	*Qwrrps.acs-5A.4*	Xgwm443-Xcfa2155	98.8	**E1**/**E3**	−1.63^***^/−1.25^***^	0.97/0.89
WCRpr	*Qwcrpr.acs-6B.1*	Xcfd13-Xwmc737	5.8	**E2**/**E3**	0.43^***^/0.50^***^	4.92/5.76
WCRps	*Qwcrps.acs-6B.1*	Xwmc341-Xbarc198	69.4	**E1**/E2	−0.85^***^/−0.99^***^	5.81/5.32
GWMS	*Qgwms.acs-2A*	Xgwm122-Xmag2150	50.4	**E1**/**E3**	−0.01^***^/− 0.01^***^	5.15/4.75

*WSC* water-soluble carbohydrate concentration, *WSCf* WSC at the anthesis stage, *WSCg* WSC at the grain-filling stage, *WSCg* WSC at the grain-filling stage, *WSCm* WSC at the maturity stage, *WRRpr* pre-anthesis WSC remobilization rate, *WRRps* post-anthesis WSC remobilization rate, *WCRpr* pre-anthesis WSC contribution rate, *WCRps* post-anthesis WSC contribution rate, *GWMS* grain weight of main spike. Site (cM), the most likely position of the putative QTL on the specific chromosome. E1 to E3 represent field trials at Anning farm station, Gansu, China (103°51′E, 36°04′N, 1600 m ASL) in 2012–2013, at Yongdeng farm station, Gansu, China (103°18′E, 36°42′N, 1950 m ASL) in 2013–2014, and at Yuzhong farm station, Gansu, China (104°07′E, 35°51′N, 1900 m ASL) in 2014–2015, respectively. *a*, the additive effect, of which a positive value indicates the Longjian 19 allele having an increasing effect on the trait value and a negative value represents the Q9086 allele having a decreasing effect on the trait value. *R*^2^(*a*) (%), the proportion of phenotypic variations explained by additive QTL. ^**^P < 0.005, ^***^P < 0.001. Water environments (E1 to E3) marked by bold typeface indicated a QTL identified in the specific environment was also interacted significantly with drought stress

All eight traits were also influenced by epistatic (*aa*) effects of the additive × additive type and the interacting effects of epistatic QEIs. A total of 88 pairs of epistatic QTLs were identified, ranging from 6 (WSCm) to 18 pairs (WCRpr) for each trait (Table S4). These nonallelic loci involved in epistasis generally showed minor *a* effects and widely distributed on all 21 chromosomes, explaining 0.90 to 11.04% of the phenotypic variance. Among them, half of pairs behaved with positive *aa* effects, indicating that the parent-type effects were higher than the recombinant-type effects. The other half, howbeit, had negative *aa* effects where recombinant-type effects were higher than parent-type effects. By contrast to other traits, WSCf and WCRps exhibited remarkable disequilibrium between the two types of *aa* effects, due to 88.9 and 72.7% of corresponding epistatic pairs with positive and negative effects, respectively. Similar to additive QTLs, most of epistatic QTLs (85 of 88, or 96.6%) were identified in single environments, whereas only three pairs, such as one pair for WSCg and two pairs for GWMS, were repeated in two environments. This suggested that epistatic QTLs expressed more environment- dependently than additive loci. When the epistatic QEIs were considered, 42 of 88 pairs (47.7%) involved in significant epistatic QEI (*aae*) effects by drought-stressed environments. For these *aae* effects, 18 pairs mostly for WSCf were positive and individually explained 1.84 to 11.42% of phenotypic variance, whereas the other 24 for all traits except WSCf were negative, individually explained 1.62 to 11.03% of phenotypic variance.

**Fig. 3:**
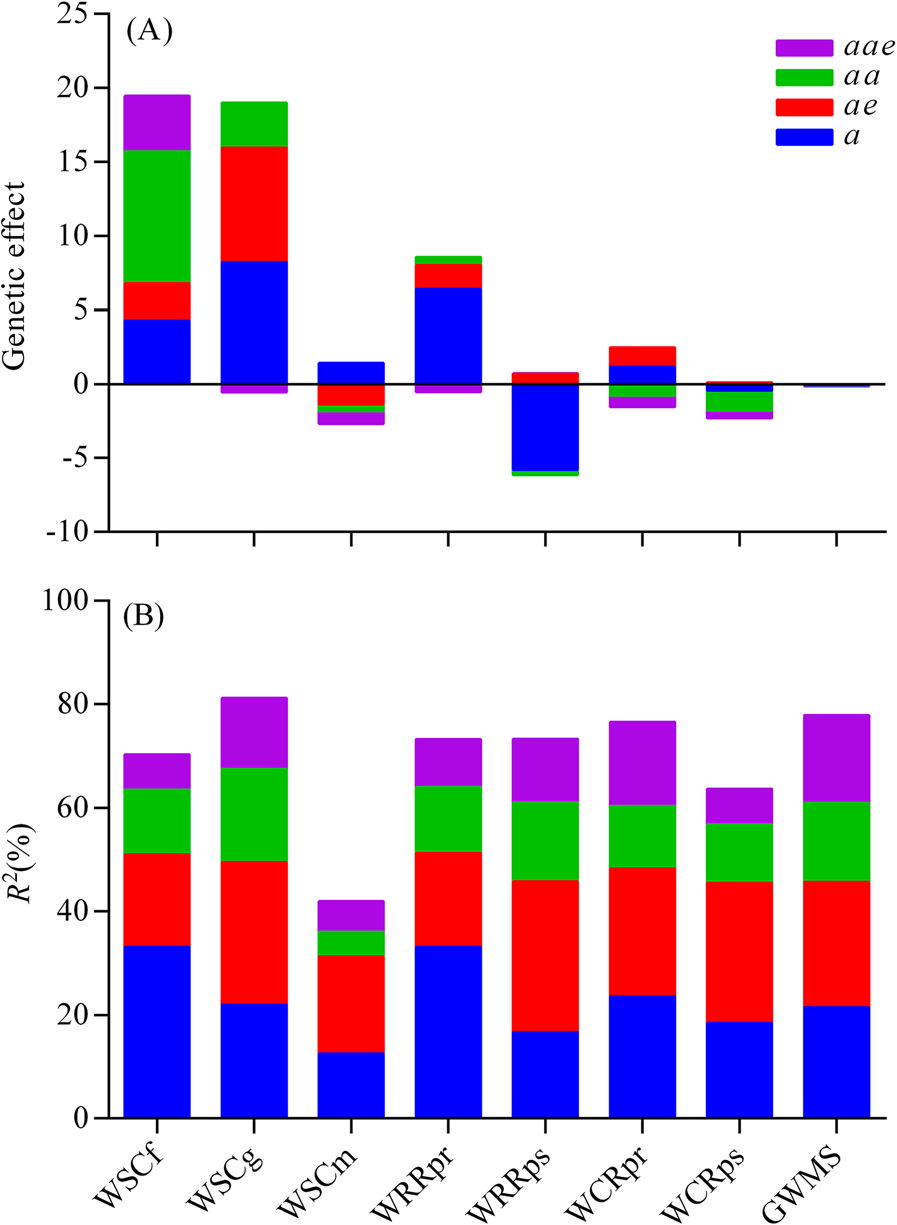
Cumulative genetic effects (**A**) and phenotypic variations (**B**) explained by different genetic components for stem WSC accumulation and remobilization in the wheat RIL population. The stem WSC-related traits evaluated herein are involved in grain weight of main spike (GWMS); water-soluble carbohydrate (WSC) concentration at the anthesis stage (WSCf), the grain-filling stage (WSCg) and the maturity stage (WSCm); WSC remobilization rate at the pre-anthesis (WRRpr) and the post-anthesis (WRRps); WSC remobilization rate at the pre-anthesis (WCRpr) and the post-anthesis (WCRps). *a*, *aa*, *ae* and *aae* represent additive effect, epistatic effect, and environmental interaction effect by additive (*ae*) and epistatic QTLs (*aae*), respectively

With regard to general effects and contributions of genetic components for all eight traits, both mean values averaged across three environmental sites significantly varied from individual genetic components and tested traits (Fig. 3). Cumulative genetic effects and interaction with drought-stressed environments behaved to increase WSCf, WSCg, WRRpr and WCRpr, but to decrease the other four traits. Comparatively, both *a* and *ae* effects for most traits were positive and more predominant in controlling phenotypic values than *aa* and *aae* effects, although *aa* or *aae* effects for WSCf, WCRps and GWMs were not ignorable (Fig. 3A). On the other hand, the phenotypic variance explained by genetic component effects also further illustrated the features of QTL expressions. In particular, cumulative phenotypic variance explained by *a* and *ae* effects accounted for 58.7% (GWMS) to 74.8% (WSCm) of total variance contributed by all genetic components, which were greater than those by both *aa* and *aae* effects (Fig. 3B). This suggested that performances of *a* and *ae* effects were highly predominant to determine the inheritance of stem WSC-related traits. In this context, some additive QTLs showed greater contributions (>10%) to phenotypic variance by *a* or *ae* effects, compared to other loci. For example, three major QTLs for WSCf and WSCg, namely *Qwscf.acs-2D*, *Qwscf.acs-3B.3* and *Qwscg.acs-6B.3*, individually explained 11.25–23.14% of the phenotypic variations by significant *a* effects. Likewise, 19 loci made greater contributions (10.04–19.86%) by significant *ae* effects to the phenotypic variations in all traits except WRRpr (Table S3).

### Chromosomal distribution of identified QTLs

The map positions of 74 additive QTLs for stem WSC-related traits had a marked tendency to cluster in 19 chromosomal regions, which formed QTL hotspots, namely, chromosomal regions shared by multiple QTLs. In the majority of QTL clusters, the closest markers of the QTLs were situated within relatively short intervals less than 10 cM and even at some specific positions on chromosomes 1B, 2A, 2B, 2D, 3B, 4B, 5A, 6A, 6B and 7A (Fig. 2, Table 3). Each chromosome harbored one (1B, 2A, 4B and 6A) to three QTL clusters (5A, 6B and 7A). For example, nine QTL clusters underlying 3–5 of the eight traits were co-located within 4–10 cM marker intervals, such as Xmag893-Xwmc44 on chromosome 1B, Xgdm87-Xbarc101 on 2B, Xksum45-Xwmc510 on 3B, and so on. Four intervals within 2 cM regions, Xmag3579-Xgwm312 on 2A, Xgdm35-Xwmc112 and Xbarc219-Xgwm349 on 2D, and Xbarc40- Xcfd2121 on 5A, harbored QTL clusters controlling 2–3 traits, respectively. Each of 13 above intervals almost covered 1–2 specific positions involving environment-specific QTLs and/or stable QTLs. For example, of seven QTLs identified in Xgwm284-Xcfd6 on 3B, two environment-specific QTLs, *Qwrrpr.acs-3B.1* and *Qwcrpr.acs-3B.1*, were co-located at the position of 46.4 cM, and two stable QTLs, *Qwscf.acs-3B.1* and *Qwscf.acs-3B.1*, and two environment-specific QTLs*, Qwscm.acs-3B.1* and *Qwcrpr.acs-3B.2*, were all located at 49.6 cM. Besides, six QTL clusters governing 2–3 traits independently located at the specific positions. Of these, three chromosomal positions at 13.4 cM on 2B, 58.4 cM on 6A and 26.7 cM on 7A harbored 2–3 environment-specific QTLs, respectively. Two positions at 98.8 cM on 5A and 5.8 cM on 6B located one stable QTL and two environment-specific QTLs, respectively. The position at 113.2 cM mapped two stable QTLs. Most of these loci were adjacent or overlapped at the left markers in flanking intervals. This indicated that these hotspot regions might carry important polygenes controlling stem WSC accumulation and remobilization, whilst a single locus governing multiple traits suggested that there could be genetic pleiotropy.

**Table 3:** Additive QTL clusters for stem WSC-related traits in the wheat RIL population

Chrom.	Flanking markers	Site (cM)	Traits	No. of QTLs
1B	Xmag893-Xwmc44	94.6–98.5	WSCf, WSCm, WRRpr, WRRps, WCRpr	6
2A	Xmag3579-Xgwm312	128.7–130.1	WSCm, WRRpr, WCRpr, WCRps	5
2B	Xwmc272-Xgwm630	13.4	WRRpr, GWMS	2
	Xgdm87-Xbarc101	52.0–59.5	WSCf, WSCm, WRRps, WCRpr	4
2D	Xgdm35-Xwmc112	2.0–3.5	WSCf, WSCg, WRRps	3
	Xbarc219-Xgwm349	89.9–91.9	WRRpr, WRRps, WCRps	3
3B	Xgwm284-Xcfd6	46.4–53.6	WSCf, WSCm, WRRpr, WRRps, WCRpr	6
	Xksum45-Xwmc510	117.3–127.3	WSCf, WSCm, WRRps	4
4B	Xgwm495-Xbarc60	76.5–84.6	WSCg, WRRps, WCRps, GWMS	5
5A	Xmag694-Xwmc705	10.4–15.8	WSCg, WRRpr, WCRps, GWMS	5
	Xbarc40-Xcfd2121	37.7–38.2	WRRpr, WRRps, GWMS	5
	Xgwm443-Xcfa2155	98.8	WSCf, WRRps, WCRps	3
6A	Xgwm570-Xwmc553	58.4	WRRpr, WCRpr	2
6B	Xcfd13-Xwmc737	5.8	WSCg, WRRps, WCRpr	3
	Xgwm193-Xwmc539	48.1–52.0	WSCg, WSCm, WRRps	3
	Xwmc341-Xwmc182	69.4–75.4	WSCg, WRRpr, WCRps	3
7A	Xbarc1034-Xwmc273	26.7	WSCf, WSCg, WCRps	3
	Xwmc603-Xwmc607	94.8–101.1	WSCm, WRRpr, WRRps,WCRps, GWMS	7
	Xbarc195-Xbarc121	113.2	WSCf, WSCm	2

*WSC* water-soluble carbohydrate concentration, *WSCf* WSC at the anthesis stage, *WSCg* WSC at the grain-filling stage, *WSCg* WSC at the grain-filling stage, *WSCm* WSC at the maturity stage, *WRRpr* pre-anthesis WSC remobilization rate, *WRRps* post-anthesis WSC remobilization rate, *WCRpr* pre-anthesis WSC contribution rate, *WCRps* post-anthesis WSC contribution rate, *GWMS* grain weight of main spike. Site (cM), the marker intervals or specific positions of QTL cluster

**Fig. 4:**
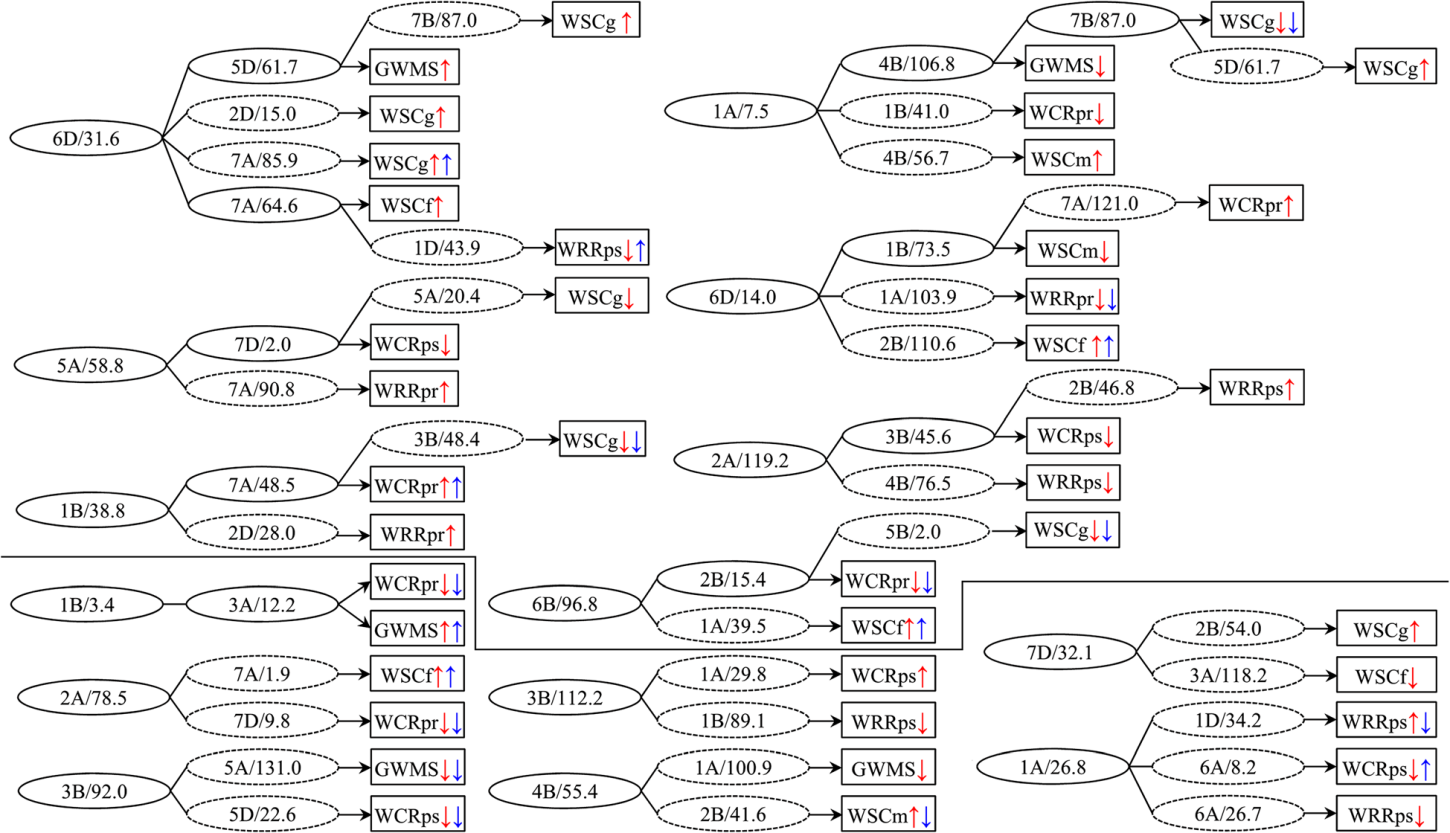
Epistatic QTL network for stem WSC-related traits in the wheat RIL population. The black dashed- and solid-line ellipses indicate one QTL interacted with another QTL and with two or more additional QTLs, respectively. The characters at the left and right of apostrophe in each black ellipse mean the specific chromosome and position for a putative QTL. The characters in each black box indicate related stem WSC-related traits, such as grain weight of main spike (GWMS); water-soluble carbohydrate (WSC) concentration at the anthesis stage (WSCf), at the grain-filling stage (WSCg) and at the maturity stage (WSCm); WSC remobilization rate at the pre-anthesis (WRRpr) and at the post-anthesis (WRRps); WSC remobilization rate at the pre-anthesis (WCRpr) and at the post-anthesis (WCRps). The red and blue arrows at the right of trait name in each black box indicate epistatic effect and epistatic QTL× environment interaction effect, respectively. Up and down arrows indicate that positive and negative genetic effects, respectively

On the other hand, nonallelic loci interacted with each other for the epistatic effects, where some loci even constituted QTL-interacting networks at different levels to realize various *aa* effects on individual traits responsive to water environments (Fig. 4). For instance, 34 nonallelic loci made up seven relatively bigger networks from four to seven-locus interactions, respectively. The other 21 loci were composed of seven smaller networks by two to three-locus interactions, respectively. In most of interaction networks, a key locus interacted with 2–4 loci to influence individual traits, of which some interactions even involved in cascade reactions to affect more traits. In addition, there existed two interacting loci concurrently regulating two different traits. This indicated that epistatic QTLs also exhibited pleiotropic functions.

## Discussions

### Phenotypic variations highly interacted with drought stress

High-level WSC reserved in stem have been suggested as the dominant carbon source for maintaining grain-filling and subsequent grain yield in wheat, particularly to offset limitations imposed on concurrent assimilation during grain development under water-deficit environments [16, 17, 19, 23]. However, previous studies have demonstrated that environmental and genotypic, and/or GEI effects, make a great influence on stem WSC levels [6, 22, 28, 29]. In the present study, the factorial ANOVA revealed that water environment and genotype predominantly affected stem WSC-related traits, whereas actions of GEIs and other interaction factors were relatively smaller (Table S1, S2). The means for most traits in the DS, except for WSCm and GWMS, were significantly higher than those in the WW. Especially in DS plants, both WRRpr and WCRpr were higher than WRRps and WCRps, while in WW plants, they were entirely opposite (Table 1). In addition, GWMS showed highly positive correlations (*r =* 0.44 to 0.75) with WSCf, WSCg, WRRpr and WCRpr under the DS, while it was positively correlated with WSCg, WRRps and WCRps (*r =* 0.55 to 0.77) under the WW (Fig. 1). This indicated that wheat stems remained higher WSC levels and greater remobilization efficiency of pre-anthesis WSC reserves for better adaptation to water-deficit environments [13–15] and partial compensation of grain filling [8, 37].

The substantial contributions from environmental and genotypic effects, along with minor effects from interaction factors, were mirrored in the moderate *h*^2^_B_ of 0.51–0.72 for these traits (Table 1). The results confirmed previous findings of larger environmental and genotypic effects on stem WSC levels with the moderate to high *h*^2^_B_ of 0.46–0.93 [22–26]. They suggested that increasing stem WSC levels via selection in breeding programs would be possible, and some field practices had been successfully performed in developing drought-tolerant wheat varieties in the UK and Australia [38, 39]. In contrast, a few of studies demonstrated that stem WSC levels were strongly influenced by GEIs, resulting in these traits showing wide fluctuations in *h*^2^_B_ (0.27–0.79) [6, 12, 28]. In this context, stem WSC levels had weak and variable correlations with grain yield, indicated that stem WSC remobilization was complex and not all of stem WSC completely contributes to the improvement of grain yield [12, 28, 37]. The most likely reason was that stem WSC reserves could be excessively consumed by plant growth and respiration [20], or highly regulated by source-sink state [40]. This suggested that direct selection of wheat breeding germplasm based on these traits was still challenging.

### Genetic components and QEI effects induced by drought stress

Phenotypic variations in stem WSC accumulation and remobilization can be essentially attributed to genetic and environmental factors, as well as their interactions [22, 28]. The intrinsic mechanism, howbeit, seems to be still limited, because these traits are inherited quantitatively and governed by polygenes [22, 28, 31–34]. On the basis of the adjusted unbiased prediction of the mixed linear model approach [41], we detected putative QTLs with *a* and *aa* effects, as well as QEI effects, for eight stem WSC-related traits using a RIL population (Table S3, S4). The result was consistent with the previous findings examining the similar traits by a DH population under two water-environmental conditions [28]. As compared with other studies involving in only the additive QTLs [32, 33], the current study revealed more comprehensive genetic information for these traits. For examples, a total of 95 additive QTLs and 88 pairs of epistatic QTLs were identified with significant main-effects (*a* and *aa*) for these traits evaluated across diverse water environments. Among them, 76.8% of additive QTLs and 47.7% of epistatic QTLs showed significant QEI effects (*ae* and *aae*) with drought-stressed environments. This indicated that epistatic QTLs expressed more environment-dependently than additive loci (Table S3, S4). Although both genetic main-effects and QEI effects were different in individual traits and water environments to various degree, the cumulative phenotypic variances explained by *a* and *ae* effects for each trait were greater than those by *aa* and *aae* effects (Fig. 3b). On the other hand, it was clear that low genetic contributions to phenotypic variance explained by *aa* effects were due to large numbers of nonallelic loci with minor *a* effects involving in epistasis (Table S4). These results supported the previous viewpoint that the stem WSC levels was mainly governed by robust *a* and *ae* effects, rather than weak epistatic effects [22]. Therefore, both *a* and *ae* effects predominantly determine the inheritance of stem WSC accumulation and remobilization in wheat.

The present study also displayed that different genetic contributions by genetic components highlighted in their diverse genetic effects induced by drought stress on stem WSC-related traits. With regard to *a* effects, most of favorable alleles (60.0–85.7%) for WSCf, WSCg, WRRpr and WCRpr showed positive effects, whereas those for WRRps (66.7%) and GWMS (90.0%) indicated negative effects. This implied that, under the DS, drought-tolerant parent Longjian19 contributed more genes expressed to increase WSCf, WSCg, WRRpr and WCRpr in the RIL progenies, whereas drought-prone parent Q9086 provided more genes to reduce WRRps and GWMS (Table S3). Similarly, WSCf and WCRps exclusively exhibited positive (88.9%) and negative (72.7%) *aa* effects, respectively (Table S4). As respects of QEI effects, most of additive QTLs (58.9%) mainly contributed positive *ae* effects for WSCf, WSCg, WRRpr and WCRpr, whereas those for epistatic QTLs (57.1%) showed negative *aae* effects for all traits except WSCf (Table S3, S4). This indicated that QEIs herein performed different *ae* and *aae* effects for individual traits. Comparatively, both *a* and *ae* effects generally dominated to increase WSCf, WSCg, WRRpr and WCRpr. The result was highly consistent with cumulative effects of genetic components for these traits (Fig. 3a), where *a* and *ae* effects for the above four traits were all positive and highly greater than *aa* and *aae* effects. As a result, it was also essentially illustrated why drought stress might improve stem WSC levels at the pre-anthesis and promote the remobilization of stem WSC reserves in the grain-filling stage, just like the results in this study (Table 1) and those in previous studies [13–15].

### QTL clusters and genetic pleiotropy

Additive QTLs for stem WSC-related traits in this study were highly concentrated in 19 chromosomal regions on chromosomes 1B, 2A, 2B, 2D, 3B, 4B, 5A, 6A, 6B and 7A (Fig. 2, Table 3, Table S3), suggesting closely linked loci controlling correlated traits. These QTL clusters were located within 10 cM of marker intervals and even at some specific positions. Each cluster almost covered 1–2 specific positions involving environment-specific QTLs and/or stable QTLs for 2–5 of the eight traits. Similarly, previous studies also reported that several genomic regions controlling stem WSC levels were co-located or adjacent by the QTLs for plant height and anthesis date [22], as well as some physiological and yield-related traits [31–34]. Compared with our early studies using the same RIL population, several QTLs for flag leaf morphology [42], leaf greenness [43], plant height [44], and thousand-grain weight [45] were shared the same marker intervals with the present QTLs for stem WSC-related traits, especially in marker intervals Xwmc272-Xgwm630 and Xgdm87-Xbarc101 on 2B, Xbarc40-Xcfd2121 on 5A, Xcfd13-Xwmc737 and Xwmc341-Xwmc182 on 6B, and Xbarc1034-Xwmc273, Xwmc603-Xwmc607 and Xbarc195-Xbarc121 on 7A (Fig. 2). This indicated that the inheritance of stem WSC-related traits, along with some critical physiological and yield-related traits, could be highly correlated with each other. However, it remains a puzzling question whether these clustered QTLs represent close linkages of multiple genes affecting different traits or have pleiotropic effects of regulatory genes that affect the related traits [46, 47]. Pleiotropy and linkage, after all, are the basis of genetic correlations [48, 49]. A feasible strategy is proposed that the dissection of pleiotropy from linkage might be resolved by increasing population sizes and marker densities, or by using overlapping substitution lines [50, 51]. However, the recent study in wheat yield-related traits showed the close linkage was difficult to differentiate from pleiotropy, and the pleiotropic architecture of the yield-syndrome was dissected more as a cause of pleiotropy rather than close linkage [48]. On the other hand, we found the interaction behaviors of nonallelic loci involving epistatic QTLs also exhibited pleiotropic effects (Fig. 4). These loci constituted QTL-interacting networks at different levels to realize various *aa* effects controlling individual traits. In these networks, a key locus interacted with another locus or several loci as cascade reactions to influence different traits. The similar result was also reported in yield-related traits of wheat [52–54], where a number of QTLs, including additive loci and/or nonallelic loci with minor *a* effects, participated in two or more epistatic interactions, making up a QTL functional network for one or more traits. This suggested that the genetic control of stem WSC and yield related traits per se was complex and, to a certain extent, reacted as part of QTL networks by the additive and epistatic pleiotropy.

### Stable QTLs and common loci compared with previous findings

For these stem WSC-related traits, we identified 13 stable QTLs that were detectable repeatedly across two of the three water environments (Table 2). Most of loci were also co-located the same chromosomal positions with environment-specific QTLs to various degree, and were adjacent or overlapped at the left markers in flanking intervals (Fig. 2, Table 3). Using a wheat microsatellite consensus map by Somers et al. [55] as a reference, some stable QTLs identified in this study were shared the similar chromosomal regions/positons with those reported earlier. For example, a stable QTL, *Qwscg.acs-2D.1*, located in the marker interval Xgwm261-Xwmc112 on 2D, was possibly the same as QTLs for stem WSC concentrations reported in several previous studies [22, 28, 29, 35], owing to proximity to Xgwm261. The locus also overlapped the position of a photoperiod gene (*Ppd-D1*) [22]. Similarly, the other two stable QTLs, *Qwrrps.acs-4B.1* for WRRps and *Qwscg.acs-4B.1* for WSCg, co-located at the chromosomal position of 76.5 cM in Xgwm495-Xgwm251, were adjacent not only to the locations of three reported QTLs for stem WSC levels [22, 31, 35], but also to the position of dwarfing gene (*Rht-B1b*) [22]. A reported locus for sucrose accumulation, mapped close to a vernalization gene (*Vrn1*) on the long arm of chromosome 5A [22], was adjacent to the position at 98.8 cM in Xgwm443-Xcfa2155 herein. This indicated that stem WSC levels could be regulated by dwarfing and development genes. Indeed, many early studies have demonstrated that genotypes with high stem WSC levels were commonly shorter, flowered earlier, and produced significantly fewer tillers than those of low WSC levels [19, 22, 27]. Besides, two QTL clusters in the intervals of Xmag3579-Xgwm312 on 2A and Xmag694-Xwmc705 on 5A overlapped or were adjacent to the locations of QTL clusters for stem WSC accumulation and remobilization detected by Yang et al. [28]. These stable and common loci, as well as closely linked molecular markers, had great potential in marker-assisted selection to improve stem WSC-related traits in wheat, especially under drought-stressed environments. Further fine-mapping these major stable QTLs and QTL regions with pleiotropic effects would advance our understanding of the underlying molecular mechanisms [56].

## Conclusions

In the present study, we found that the inheritance of stem WSC-related traits in wheat was predominantly governed by additive and QEI effects, indicating a moderate heritability. Most of additive QEIs associated with drought-stressed environments functioned positive regulation on stem WSC accumulation and remobilization efficiency at pre-anthesis. QTL cluster regions identified were suggestive of tight linkage or pleiotropy in the inheritance of these traits. Some stable and common loci, as well as closely linked molecular markers, had great potential in marker-assisted selection to improve stem WSC-related traits in wheat, especially under drought-stressed environments.

## Methods

### Plant materials and field trials

A set of the hexaploid wheat RIL population composed of 120 lines were used in this study. The population was derived from a cross between two Chinese winter wheat cultivars, Longjian 19 and Q9086, and its development is described in our previous studies [42, 43]. The male parent Longjian 19 is an elite drought-tolerant cultivar widely grown in rainfed areas (300–500 mm annual rainfall) in northwestern China. The female parent Q9086 is a high-yielding cultivar suitable for cultivation under conditions of sufficient water and high fertility, but is prone to premature senescence under terminal drought stress. Two parents differ significantly from several physiological and agronomical traits under drought stress [42–45, 57].

Field trials were carried out at Anning farm station, Gansu, China (103°51′E, 36°04′N, 1600 m ASL) in 2012–2013 (E1), at Yongdeng farm station, Gansu, China (103°18′E, 36°42′N, 1950 m ASL) in 2013–2014 (E2), and at Yuzhong farm station, Gansu, China (104°07′E, 35°51′N, 1900 m ASL) in 2014–2015 (E3), respectively. These sites belong to the typical inland arid areas in Northwest China, where the annual rainfall is below 400 mm with more than 1500 mm of the annual evaporation capacity. All of progenies and parents were sown in late September and harvested in early July of the following year. Field trials at each site were managed under DS and WW sections. The DS plots were equivalent to rainfed condition with the rainfall of 115 (E1), 80 (E2) and 142 mm (E3) in each growing season, respectively, whereas the WW ones were irrigated with 75 mm water supply at the heading (Zadoks 55) and grain filling (Zadoks 71) stages, respectively. Herein growth stages were recorded using decimal codes described by Zadoks et al. [58], and the specific dates of major growth stages were shown in Table S5. Field experimental designs were randomized complete blocks with three replications. Each plot was 1 m long with 6 rows spaced 20 cm apart. Approximately 60 seeds per row were sown. Field management aspects followed the local practices during wheat production, as described in our previous study [42].

### Sampling and assays of stem WSC

In each plot, five main shoots with the same anthesis date were randomly selected as samples. They were cut at the soil surface at three phenological stages, viz., anthesis (Zadoks 60), grain filling (Zadoks 71), and grain physiological maturity (Zadoks 92). Leaves and spikes were removed and the main stems retaining only the culms were put into liquid nitrogen and were dehydrated in a refrigerated-vacuum evaporator at 8.1 kPa air pressure and − 60 °C for 24 h. Dehydrated samples were continuously treated at 105 °C for 20 min and further dried at 80 °C until a constant dry weight available. The dry weight (DW) for main stems for each genotype was measured as sampled at the anthesis (SDWf), grain filling (SDWg), and maturity stages (SDWm), respectively. The corresponding main spikes sampled at the maturity stage were collected to dry for each genotype, and grains were threshed and weighed to obtain GWMS.

Dry stem samples were chopped into pieces of about 1–2 mm in length. Stem WSC was extracted according to a modified procedure described by Wardlaw and Willenbrink [9]. Extractions were performed with 0.1 g dry material for each sample with three replications. Samples were incubated in 40 ml hot ddH_2_O (90 °C) for 1 h. The fractions were filtered and centrifuged at 10000 g for 5 min. The supernatant was transferred to volumetric flasks (50 ml) and added ddH_2_O to 50 ml. Total amounts of stem WSC (mg. g^− 1^ DW) were determined as fructose equivalents using the anthrone colorimetric assay [59] at 620 nm on a TU-1810 spectrophotometer (Bejing Persee Co., China). Three independent assays were conducted on each sample. The mean values were used to estimate WSCf, WSCg and WSCm, respectively.

On the assay data available, stem WSC remobilization and its efficiency were calculated as pre-and post-anthesis ones. Of these, WRRpr (%) and WRRps (%) were evaluated as percentage by (WSCf × SDWf - WSCm × SDWm)/(WSCf × SDWf) × 100% and by (WSCg × SDWg - WSCf × SDWf)/(WSCg × SDWg) × 100%, respectively. WCRpr (%) and WCRps (%) were estimated as percentage by (WSCf × SDWf - WSCm × SDWm)/(GWMS × 1000) × 100% and by (WSCg × SDWg - WSCf × SDWf)/(GWMS × 1000) × 100%, respectively.

### Data analysis

Statistical analysis was implemented using the SPSS version 18.0 statistical package (California State University Information Services, Los Angeles, CA, USA), and *P* values less than 0.05 were significant. The ANOVA based on combined linear mixed model was used to estimate the total and residual variances among RIL progenies and parents for each stem WSC-related trait under different water environments. Basic statistics and Pearson correlation analysis were performed on the mean data averaged across three environments under DS and WW conditions. The *h*^2^_B_ was calculated for each trait using the formula proposed by Toker [60]. Here, *h*^2^_B_ = *σ*_*g*_^2^/(*σ*_*g*_^2^ + *σ*_*ge*_^2^/*r* + *σ*_*e*_^2^/*re*), where *σ*_*g*_^2^, *σ*_*ge*_^2^ and *σ*_*e*_^2^ were estimates of genotype, genotype × environment interaction and residual error variances, respectively, and *e* and *r* were the numbers of environments and replicates per environment, respectively.

The genetic linkage map was developed using the RIL population, consisting of 524 simple sequence repeats (SSR) marker loci covering 2266.7 cM with an average distance of 4.3 cM between adjacent markers [42–45]. QTL analysis was performed by the mixed linear model mapping [41], using the Windows version computer program QTLNetwork 2.0 [61]. Composite interval analysis was carried out by forward-backward stepwise, multiple linear regression with a probability into and out of the model of 0.05 and a window size set at 10 cM. Significant thresholds for QTL detection were calculated for each dataset using 1000 permutations and a genome-wide error rate of 0.05. The final genetic model incorporated significant main-effects of *a* and *aa*, as well as QEI effects of *ae* and *aae*. In such a model, all possible pairs of markers were scanned and tested. The locations of individual QTLs were drawn on genetic maps using the software of MapChart 2.1 [62].

## Supplementary information

**Additional file 1: Table S1.** Mean squares of analysis of variance (ANOVA) for stem WSC-related traits in parents of the wheat RIL population. **Table S2.** Mean squares of analysis of variance (ANOVA) for stem WSC-related traits in the wheat RIL population. **Table S3.** Additive and interaction effects of QTL × environment of QTLs identified for stem WSC-related traits in the wheat RIL population. **Table S4.** Epistatic effects and interacting effects of epistatic QTL × environment of QTLs identified for stem WSC-related traits in the wheat RIL population. **Table S5.** The specific dates of major growth stages recorded in the wheat RIL population under drought-stressed (DS) and well-watered (WW) conditions in different environments.

## Data Availability

The data sets supporting the results of this article are included in this manuscript and its additional information files.

## Abbreviations

*a*: additive effect
*aa*: epistatic effect
*aae*: interaction effect of epistatic QTL× water environment
*ae*: interaction effect of additive QTL× water environment
ANOVA: Analysis of variance
DH: Doubled-haploid
DS: Drought stress
DW: Dry weight
GEI: Genotype × environment interaction
GWMS: Grain weight of main spike
*h*^2^_B_: broad-sense heritability
QEI: QTL× water environmental interaction
QTL: Quantitative trait locus
*r*: correlation coefficient
RIL: Recombinant inbred lines
SDWf: Stem dry weight at the anthesis
SDWg: Stem dry weight at the grain filling
SDWm: Stem dry weight at the maturity stages
SSR: Simple sequence repeats
WCRpr: Stem WSC contribution rates to GWMS at pre-anthesis
WCRps: Stem WSC contribution rates to GWMS at post-anthesis
WRRpr: Stem WSC remobilization rates at pre-anthesis
WRRps: Stem WSC remobilization rates at post-anthesis
WSC: Water soluble carbohydrates
WSCf: Stem WSC concentration at the anthesis stage
WSCg: Stem WSC concentration at the grain filling stage
WSCm: Stem WSC concentration at the maturity stage
WUE: Water-use efficiency
WW: Well-watered
