# Supplementary material for: Genetic dissection of stem WSC accumulation and remobilization in wheat (Triticum aestivum L.) under terminal drought stress
Source: BMC Genet. 2020 Apr 29;21:50. doi: 10.1186/s12863-020-00855-1 (PMC7191701; doi:10.1186/s12863-020-00855-1)
Supplement: Supplementary file 1 — Additional file 1: Table S1. Mean squares of analysis of variance (ANOVA) for stem WSC-related traits in parents of the wheat RIL population. Table S2. Mean squares of analysis of variance (ANOVA) for stem WSC-related traits in the wheat RIL population. Table S3. Additive and interaction effects of QTL × environment of QTLs identified for stem WSC-related traits in the wheat RIL population. Table S4. Epistatic effects and interacting effects of epistatic QTL × environment of QTLs identified for stem WSC-related traits in the wheat RIL population. Table S5. The specific dates of major growth stages recorded in the wheat RIL population under drought-stressed (DS) and well-watered (WW) conditions in different environments. [file 12863_2020_855_MOESM1_ESM.docx]

**Table S1 Mean squares of analysis of variance (ANOVA) for stem WSC-related traits in parents of the wheat RIL population**

| Variable | WSCf  (mg.g^-1^ DW) | WSCg  (mg.g^-1^ DW) | WSCm  (mg.g^-1^ DW) | WRRpr  (%) | WRRps  (%) | WCRpr  (%) | WCRps  (%) | GWMS (g) |
| --- | --- | --- | --- | --- | --- | --- | --- | --- |
| Site (S) | 592.32^**^ | 635.10^**^ | 830.35^**^ | 129.35^**^ | 500.06^**^ | 198.10^**^ | 176.94^**^ | 0.03^**^ |
| Water (W) | 5987.14^**^ | 14972.46^**^ | 22543.82^**^ | 18764.10^**^ | 951.05^**^ | 674.51^**^ | 411.96^**^ | 1.04^**^ |
| Genotype (G) | 1886.40^**^ | 10407.01^**^ | 3711.32^**^ | 944.12^**^ | 891.35^**^ | 258.42^**^ | 374.08^**^ | 0.37^**^ |
| S×W | 638.28^**^ | 50.44 | 350.28^**^ | 63.19^**^ | 251.98^**^ | 271.35^**^ | 148.49^**^ | 0.02^*^ |
| S×G | 30.76^**^ | 71.77 | 182.82^**^ | 43.28^**^ | 81.10^**^ | 7.32^**^ | 10.60^**^ | 0.01 |
| W×G | 8.04 | 376.36^**^ | 1023.18^**^ | 189.57^**^ | 85.95^**^ | 20.16^**^ | 58.00^**^ | 0.02^*^ |
| S×W×G | 83.75^**^ | 42.42 | 300.55^**^ | 68.80^**^ | 192.29^**^ | 16.60^**^ | 58.46^**^ | 0.01 |
| Error | 3.57 | 22.00 | 14.12 | 5.31 | 4.22 | 0.64 | 1.35 | 0.01 |

WSC, water-soluble carbohydrate concentration; WSCf, WSC at the anthesis stage; WSCg, WSC at the grain-filling stage; WSCg, WSC at the grain-filling stage; WSCm, WSC at the maturity stage; WRRpr, pre-anthesis WSC remobilization rate; WRRps, post-anthesis WSC remobilization rate; WCRpr, pre-anthesis WSC contribution rate; WCRps, post-anthesis WSC contribution rate; GWMS, grain weight of main spike. DS, drought-stressed condition; WW, well-watered condition. DW, dry weight. ^*^*P*＜0.05, ^**^ *P*＜0.01.

**Table S2 Mean squares of analysis of variance (ANOVA) for stem WSC-related traits in the wheat RIL population**

| Variable | WSCf  (mg.g^-1^ DW) | WSCg  (mg.g^-1^ DW) | WSCm  (mg.g^-1^ DW) | WRRpr  (%) | WRRps  (%) | WCRpr  (%) | WCRps  (%) | GWMS (g) |
| --- | --- | --- | --- | --- | --- | --- | --- | --- |
| Site (S) | 101841.81^**^ | 52390.00^**^ | 62057.85^**^ | 4258.69^**^ | 26012.98^**^ | 401.21^**^ | 5396.24^**^ | 0.54^**^ |
| Water (W) | 137867.45^**^ | 196069.81^**^ | 774800.44^**^ | 1032592.31^**^ | 1361.10^**^ | 65112.00^**^ | 288.30^**^ | 52.23^**^ |
| Genotype (G) | 1772.68^**^ | 4209.75^**^ | 888.70^**^ | 285.57^**^ | 487.55^**^ | 78.14^**^ | 117.85^**^ | 0.13^**^ |
| S×W | 4083.46^**^ | 7849.01^**^ | 19897.98 | 4755.56^**^ | 6444.46^**^ | 396.68^**^ | 1541.65^**^ | 1.57^**^ |
| S×G | 671.23^**^ | 1350.62^**^ | 348.34^**^ | 120.50^**^ | 232.25^**^ | 30.41^*^ | 47.31^**^ | 0.06^**^ |
| W×G | 1335.77^**^ | 3300.07^**^ | 863.96^**^ | 253.21^**^ | 522.90^**^ | 67.29^**^ | 95.43^**^ | 0.11^**^ |
| S×W×G | 657.75^**^ | 919.59^**^ | 313.90^**^ | 108.56^**^ | 202.91^**^ | 27.95^*^ | 37.20^*^ | 0.04^*^ |
| Error | 61.43 | 52.88 | 38.22 | 24.66 | 30.29 | 2.32 | 5.76 | 0.01 |

WSC, water-soluble carbohydrate concentration; WSCf, WSC at the anthesis stage; WSCg, WSC at the grain-filling stage; WSCg, WSC at the grain-filling stage; WSCm, WSC at the maturity stage; WRRpr, pre-anthesis WSC remobilization rate; WRRps, post-anthesis WSC remobilization rate; WCRpr, pre-anthesis WSC contribution rate; WCRps, post-anthesis WSC contribution rate; GWMS, grain weight of main spike. DS, drought-stressed condition; WW, well-watered condition. DW, dry weight. ^*^*P*＜0.05, ^**^ *P*＜0.01.

**Table S3 Additive and interaction effects of QTL × water environment of QTLs identified for stem WSC-related traits in the wheat RIL population**

| Trait | QTL | Flanking markers | Site (cM) | Environ. | *a* | *R*^2^(*a*)% | *ae* | *R*^2^(*ae*)% |
| --- | --- | --- | --- | --- | --- | --- | --- | --- |
| WSCf | *Qwscf.acs-1B.1* | Xmag893-Xwmc830 | 94.6 | E1 | -2.48^***^ | 3.40 | 3.46^***^ | 13.16 |
|  | *Qwscf.acs-2B.1* | Xgdm87-Xgwm388 | 52.0 | E1 | 1.32^**^ | 2.10 |  |  |
|  | *Qwscf.acs-2B.2* | Xgwm501-Xmag956 | 93.3 | E1 | -2.14^***^ | 2.52 |  |  |
|  | *Qwscf.acs-2D* | Xgdm35-Xgwm261 | 2.0 | E2 | 4.17^***^ | 23.14 | 3.63^***^ | 17.86 |
|  | *Qwscf.acs-3B.1* | Xpsp3112-Xgwm72 | 49.6 | E1 | -3.55^***^ | 6.96 | -3.03^***^ | 10.04 |
|  |  |  |  | E3 | -3.48^***^ | 8.09 |  |  |
|  | *Qwscf.acs-3B.2* | Xksum45-Xwmc510 | 117.3 | E1 | 1.81^**^ | 3.26 |  |  |
|  | *Qwscf.acs-3B.3* | Xksum45-Xwmc510 | 127.3 | E3 | 5.26^***^ | 18.66 |  |  |
|  | *Qwscf.acs-4A* | Xksum51-Xgwm637 | 75.7 | E1 | 4.07^***^ | 9.14 | 3.44^***^ | 12.51 |
|  |  |  |  | E3 | 3.77^***^ | 9.58 |  |  |
|  | *Qwscf.acs-5A.1* | Xgwm443-Xcfa2155 | 98.8 | E2 | -1.35^**^ | 2.48 |  |  |
|  | *Qwscf.acs-7A.1* | Xbarc1034-Xwmc273 | 26.7 | E3 | 1.72^**^ | 2.64 |  |  |
|  | *Qwscf.acs-7A.2* | Xbarc195-Xbarc121 | 113.2 | E2 | 1.59^***^ | 3.42 |  |  |
|  |  |  |  | E3 | 2.12^***^ | 4.12 |  |  |
| WSCg | *Qwscg.acs-2D.1* | Xgwm261-Xwmc112 | 3.5 | E1 | -2.45^***^ | 3.92 | 3.68^***^ | 8.81 |
|  |  |  |  | E2 | -3.45^***^ | 4.62 |  |  |
|  | *Qwscg.acs-4B.1* | Xgwm495-Xgwm251 | 76.5 | E2 | -3.55^***^ | 4.91 |  |  |
|  |  |  |  | E3 | -2.36^***^ | 3.08 | 2.93^***^ | 5.34 |
|  | *Qwscg.acs-4B.2* | Xwmc349-Xwmc413 | 82.1 | E2 | 3.20^***^ | 3.99 |  |  |
|  | *Qwscg.acs-5A.1* | Xmag694-Xgwm415 | 10.4 | E2 | 2.26^***^ | 2.97 | -3.21^***^ | 6.26 |
|  |  |  |  | E3 | 3.32^***^ | 3.83 | -4.42^***^ | 13.53 |
|  | *Qwscg.acs-6A* | Xwmc684-Xbarc201 | 36.9 | E2 | -3.30^***^ | 4.25 |  |  |
|  | *Qwscg.acs-6B.1* | Xcfd13-Xwmc737 | 5.8 | E1 | -4.22^***^ | 9.14 | 3.84^***^ | 15.14 |
|  | *Qwscg.acs-6B.2* | Xgwm193-Xgwm361 | 48.1 | E3 | 4.42^***^ | 6.77 | -3.27^***^ | 7.35 |
|  | *Qwscg.acs-6B.3* | Xbarc198-Xwmc182 | 75.4 | E3 | -5.88^***^ | 11.25 | 3.40^***^ | 8.01 |
|  | *Qwscg.acs-7A.1* | Xbarc1034-Xwmc273 | 26.7 | E3 | 4.58^***^ | 7.26 | -5.11^***^ | 18.09 |
| WSCm | *Qwscm.acs-1B.1* | Xwmc830-Xwmc44 | 96.5 | E3 | 1.74^**^ | 1.74 | 1.67^**^ | 6.36 |
|  | *Qwscm.acs-2A* | Xmag3579-Xcfd168 | 128.7 | E3 | -1.94^***^ | 4.32 |  |  |
|  | *Qwscm.acs-2B.1* | Xgdm87-Xgwm388 | 52.0 | E2 | -1.90^***^ | 1.50 | 1.96^**^ | 3.24 |
|  | *Qwscm.acs-3B.1* | Xpsp3112-Xgwm72 | 49.6 | E1 | -1.70^**^ | 2.58 |  |  |
|  | *Qwscm.acs-3B.2* | Xksum45-Xwmc510 | 119.3 | E3 | 2.53^***^ | 7.32 | -2.95^***^ | 19.86 |
|  | *Qwscm.acs-6B* | Xgwm70-Xwmc539 | 52.0 | E2 | -1.88^***^ | 1.50 |  |  |
|  | *Qwscm.acs-7A.1* | Xwmc116-Xksum153 | 95.5 | E3 | 2.34^***^ | 6.30 | -2.21^***^ | 11.22 |
|  | *Qwscm.acs-7A.2* | Xbarc195-Xbarc121 | 113.2 | E1 | 2.29^***^ | 3.48 |  |  |
|  |  |  |  | E3 | 2.80^***^ | 8.94 | -2.63^***^ | 15.78 |
| WRRpr | *Qwrrpr.acs-1A.1* | Xgwm633-Xgwm164 | 37.5 | E3 | 1.57^***^ | 6.24 |  |  |
|  | *Qwrrpr.acs-1B.1* | Xwmc830-Xwmc44 | 98.5 | E3 | 1.09^***^ | 6.84 | 1.07^***^ | 3.90 |
|  | *Qwrrpr.acs-2A.1* | Xbarc208-Xgwm515 | 74.1 | E3 | 1.86^***^ | 8.40 | 1.70^***^ | 9.82 |
|  | *Qwrrpr.acs-2A.2* | Xcfd168-Xgwm312 | 130.1 | E3 | -1.24^***^ | 7.02 | -1.36^***^ | 6.30 |
|  | *Qwrrpr.acs-2B.1* | Xwmc272-Xgwm630 | 13.4 | E3 | 1.23^***^ | 7.43 | 1.16^***^ | 4.65 |
|  | *Qwrrpr.acs-2D.1* | Xbarc219-Xgwm349 | 91.9 | E2 | -1.39^***^ | 6.78 | -1.71^***^ | 6.17 |
|  | *Qwrrpr.acs-3B.1* | Xgwm284-Xpsp3112 | 46.4 | E1 | 2.95^***^ | 6.54 |  |  |
|  | *Qwrrpr.acs-5A.1* | Xbarc165-Xwmc705 | 15.8 | E3 | 1.88^***^ | 6.42 | -0.70^***^ | 1.65 |
|  | *Qwrrpr.acs-5A.2* | Xbarc40-Xbarc141 | 37.7 | E3 | 1.11^***^ | 6.81 | 1.30^***^ | 5.70 |
|  | *Qwrrpr.acs-5A.3* | Xbarc141-Xcfd2121 | 38.2 | E1 | 1.93^***^ | 6.48 | 1.25^***^ | 4.34 |
|  | *Qwrrpr.acs-6A* | Xgwm570-Xwmc553 | 58.4 | E3 | 1.95^***^ | 6.52 | 1.07^***^ | 3.71 |
|  | *Qwrrpr.acs-6B.1* | Xmag1266-Xmag962 | 36.3 | E3 | 1.64^***^ | 6.30 | 1.12^***^ | 1.65 |
|  | *Qwrrpr.acs-6B.2* | Xwmc341-Xbarc198 | 69.4 | E1 | 1.68^***^ | 5.49 |  |  |
|  |  |  |  | E3 | 1.96^***^ | 6.66 | 0.91^***^ | 2.85 |
|  | *Qwrrpr.acs-7A.1* | Xwmc116-Xksum153 | 95.5 | E3 | 1.01^***^ | 5.58 | -1.04^***^ | 3.75 |
| WRRps | *Qwrrps.acs-1A.1* | Xgwm164-Xcfd59 | 53.5 | E1 | -1.18^***^ | 1.09 | -1.21^***^ | 2.28 |
|  | *Qwrrps.acs-1B.1* | Xwmc830-Xwmc44 | 96.5 | E1 | 1.24^***^ | 1.20 | -1.29^***^ | 2.61 |
|  | *Qwrrps.acs-1B.2* | Xmag893-Xwmc830 | 94.6 | E3 | -2.50^***^ | 3.55 | -2.26^***^ | 5.84 |
|  | *Qwrrps.acs-2A.1* | Xgwm515-Xwmc644 | 77.1 | E1 | -1.35^***^ | 1.42 | -1.48^***^ | 3.44 |
|  | *Qwrrps.acs-2B.1* | Xgdm87-Xgwm388 | 52.0 | E1 | -1.76^***^ | 1.12 | -0.72^***^ | 0.81 |
|  | *Qwrrps.acs-2D.1* | Xgwm261-Xwmc112 | 3.5 | E2 | -2.02^***^ | 3.71 | 2.26^***^ | 9.32 |
|  | *Qwrrps.acs-2D.2* | Xbarc219-Xgwm349 | 89.9 | E2 | -2.11^***^ | 4.05 | -1.90^***^ | 6.56 |
|  | *Qwrrps.acs-3B.1* | Xgwm72-Xcfd6 | 53.6 | E3 | -1.72^***^ | 1.69 | -1.92^***^ | 4.20 |
|  | *Qwrrps.acs-3B.2* | Xksum45-Xwmc510 | 117.3 | E1 | 1.15^***^ | 1.04 | 1.00^***^ | 1.56 |
|  | *Qwrrps.acs-4A.1* | Xgwm601-Xcfa2256 | 13.1 | E3 | 3.04^***^ | 5.25 | 3.10^***^ | 10.97 |
|  | *Qwrrps.acs-4A.2* | Xgwm613-Xmag3733 | 55.5 | E3 | -1.43^***^ | 1.16 | -1.50^***^ | 2.57 |
|  | *Qwrrps.acs-4B.1* | Xgwm495-Xgwm251 | 76.5 | E1 | -1.50^***^ | 1.75 | 1.16^***^ | 2.11 |
|  |  |  |  | E2 | -3.05^***^ | 5.31 | 2.70^***^ | 8.31 |
|  | *Qwrrps.acs-5A.1* | Xbarc40-Xbarc141 | 37.7 | E1 | -1.31^***^ | 1.34 | 1.31^***^ | 2.70 |
|  | *Qwrrps.acs-5A.2* | Xbarc141-Xcfd2121 | 38.2 | E2 | -2.24^***^ | 4.55 | 1.79^***^ | 5.87 |
|  | *Qwrrps.acs-5A.3* | Xcfd2121-Xgwm639 | 47.1 | E1 | -1.72^***^ | 2.31 | -1.69^***^ | 4.48 |
|  | *Qwrrps.acs-5A.4* | Xgwm443-Xcfa2155 | 98.8 | E1 | -1.63^***^ | 0.97 | 0.80^***^ | 0.99 |
|  |  |  |  | E3 | -1.25^***^ | 0.89 | 1.39^***^ | 2.20 |
|  | *Qwrrps.acs-6B.1* | Xcfd13-Xwmc737 | 5.8 | E1 | -1.90^***^ | 1.30 | -0.97^***^ | 1.46 |
|  | *Qwrrps.acs-6B.2* | Xgwm70-Xwmc539 | 52.0 | E1 | 1.03^***^ | 0.83 | 0.96^***^ | 1.44 |
|  | *Qwrrps.acs-6B.3* | Xmag2276-Xbarc79 | 87.5 | E2 | 1.47^***^ | 1.98 |  |  |
|  | *Qwrrps.acs-7A.1* | Xwmc603-Xwmc116 | 94.8 | E3 | 2.06^***^ | 2.41 | 2.24^***^ | 5.70 |
|  | *Qwrrps.acs-7A.2* | Xwmc116-Xksum153 | 95.5 | E1 | 1.22^***^ | 1.17 | 1.05^***^ | 1.74 |
| WCRpr | *Qwcrpr.acs-1A.1* | Xksum104-Xmag1022 | 22.8 | E1 | -0.39^**^ | 7.40 |  |  |
|  | *Qwcrpr.acs-1B.1* | Xwmc830-Xwmc44 | 96.5 | E1 | 0.86^***^ | 7.64 | 0.88^***^ | 7.64 |
|  | *Qwcrpr.acs-2A.1* | Xmag3579-Xcfd168 | 128.7 | E1 | 0.90^***^ | 8.00 | 0.87^***^ | 7.41 |
|  | *Qwcrpr.acs-2A.2* | Xcfd168-Xgwm312 | 130.1 | E3 | 1.04^***^ | 7.72 | 0.96^***^ | 17.36 |
|  | *Qwcrpr.acs-2B.1* | Xwmc223-Xbarc101 | 59.5 | E2 | 1.05^**^ | 5.36 | -0.25^***^ | 4.60 |
|  | *Qwcrpr.acs-3B.1* | Xgwm284-Xpsp3112 | 46.4 | E3 | -0.70^***^ | 7.52 | 0.62^***^ | 9.52 |
|  | *Qwcrpr.acs-3B.2* | Xpsp3112-Xgwm72 | 49.6 | E1 | -0.50^***^ | 5.24 | -0.56^***^ | 7.12 |
|  | *Qwcrpr.acs-3B.3* | Xksum45-Xwmc510 | 133.3 | E3 | 0.52^***^ | 5.96 | 0.47^***^ | 7.24 |
|  | *Qwcrpr.acs-6A.1* | Xgwm570-Xwmc553 | 58.4 | E3 | -0.26^**^ | 5.24 |  |  |
|  | *Qwcrpr.acs-6B.1* | Xcfd13-Xwmc737 | 5.8 | E2 | 0.43^***^ | 4.92 | 0.47^***^ | 6.12 |
|  |  |  |  | E3 | 0.50^***^ | 5.76 | 0.46^***^ | 7.08 |
| WCRps | *Qwcrps.acs-2A.1* | Xmag3579-Xcfd168 | 128.7 | E1 | -0.59^***^ | 2.77 | -0.53^***^ | 4.48 |
|  | *Qwcrps.acs-2D.1* | Xbarc219-Xgwm349 | 89.9 | E2 | -0.48^***^ | 1.91 | -0.39^***^ | 2.52 |
|  | *Qwcrps.acs-4B* | Xwmc413-Xbarc60 | 84.6 | E1 | 0.49^***^ | 1.92 | 0.54^***^ | 4.70 |
|  | *Qwcrps.acs-5A.1* | Xmag694-Xgwm415 | 10.4 | E3 | -1.30^***^ | 9.15 | -0.97^***^ | 10.13 |
|  | *Qwcrps.acs-5A.2* | Xgwm443-Xcfa2155 | 98.8 | E3 | -1.05^***^ | 6.00 | -1.07^***^ | 12.45 |
|  | *Qwcrps.acs-6A.1* | Xgwm169-Xwmc580 | 69.8 | E1 | 0.86^***^ | 5.89 | -0.54^***^ | 4.67 |
|  | *Qwcrps.acs-6A.2* | Xwmc580-Xksum255 | 74.3 | E3 | 0.62^***^ | 2.10 |  |  |
|  | *Qwcrps.acs-6B.1* | Xwmc341-Xbarc198 | 69.4 | E1 | -0.85^***^ | 5.81 | 0.99^***^ | 15.62 |
|  |  |  |  | E2 | -0.99^***^ | 5.32 |  |  |
|  | *Qwcrps.acs-7A.1* | Xbarc1034-Xwmc273 | 26.7 | E3 | 0.79^***^ | 3.35 | 0.84^***^ | 7.54 |
|  | *Qwcrps.acs-7A.2* | Xmag3023-Xwmc596 | 38.9 | E2 | 0.98^***^ | 7.88 | 0.98^***^ | 15.85 |
|  | *Qwcrps.acs-7A.3* | Xwmc603-Xwmc116 | 94.8 | E3 | 0.58^***^ | 1.81 |  |  |
|  | *Qwcrps.acs-7A.4* | Xksum153-Xwmc607 | 100.1 | E1 | -0.43^***^ | 1.45 | 0.46^***^ | 3.31 |
| GWMS | *Qgwms.acs-1A.1* | Xbarc240-Xpsp2999 | 70.2 | E2 | -0.02^***^ | 5.80 |  |  |
|  | *Qgwms.acs-2A* | Xgwm122-Xmag2150 | 50.4 | E1 | -0.01^***^ | 5.15 | 0.03^***^ | 11.45 |
|  |  |  |  | E3 | -0.01^***^ | 4.75 | 0.02^***^ | 4.85 |
|  | *Qgwms.acs-2B.1* | Xwmc272-Xgwm630 | 13.4 | E2 | -0.02^***^ | 3.62 |  |  |
|  | *Qgwms.acs-2B.2* | Xgwm547-Xmag4281 | 91.5 | E2 | 0.02^***^ | 8.35 | 0.02^***^ | 7.40 |
|  | *Qgwms.acs-2D* | Xmag1280-Xgwm157 | 32.5 | E2 | -0.02^***^ | 3.25 |  |  |
|  | *Qgwms.acs-4B.1* | Xwmc349-Xwmc413 | 82.1 | E2 | -0.02^***^ | 6.39 | -0.02^***^ | 12.72 |
|  | *Qgwms.acs-5A.1* | Xgwm415-Xbarc165 | 14.3 | E1 | -0.03^***^ | 7.35 | -0.03^***^ | 9.26 |
|  | *Qgwms.acs-5A.2* | Xbarc165-Xwmc705 | 15.8 | E3 | -0.03^***^ | 4.45 | -0.03^***^ | 7.60 |
|  | *Qgwms.acs-5A.3* | Xbarc141-Xcfd2121 | 38.2 | E2 | -0.02^***^ | 8.35 | -0.02^***^ | 6.41 |
|  | *Qgwms.acs-7A.1* | Xwmc603-Xwmc116 | 94.8 | E2 | -0.02^***^ | 7.18 | 0.02^***^ | 12.75 |

WSC, water-soluble carbohydrate concentration; WSCf, WSC at the anthesis stage; WSCg, WSC at the grain-filling stage; WSCg, WSC at the grain-filling stage; WSCm, WSC at the maturity stage; WRRpr, pre-anthesis WSC remobilization rate; WRRps, post-anthesis WSC remobilization rate; WCRpr, pre-anthesis WSC contribution rate; WCRps, post-anthesis WSC contribution rate; GWMS, grain weight of main spike. Site (cM), the most likely position of the putative QTL on the specific chromosome. E1 to E3 represent field trials at Anning farm station, Gansu, China (103º51'E, 36º04'N, 1600 m ASL) in 2012-2013, at Yongdeng farm station, Gansu, China (103º18'E, 36º42'N, 1950 m ASL) in 2013-2014, and at Yuzhong farm station, Gansu, China (104º07'E, 35º51'N, 1900 m ASL) in 2014-2015, respectively. *a*, the additive effect, of which a positive value indicates the Longjian 19 allele having an increasing effect on the trait value and a negative value represents the Q9086 allele having a decreasing effect on the trait value. *R*^2^(*a*) (%), the proportion of phenotypic variations explained by additive QTL. *ae*, the additive QTL × environment interaction effects, of which a positive value indicates *ae* effect having an increasing effect on the trait value in DS conditions and a negative value means *ae* effect having a decreasing effect on the trait value in WW conditions. *R*^2^(*ae*)(%), the phenotypic variations explained by additive QTL×environment interaction. ^**^*P*＜0.005, ^***^*P*＜0.001.

**Table S4 Epistatic effects and interacting effects of epistatic QTL × water environment of QTLs identified for stem WSC-related traits in the wheat RIL population**

| Trait | *QTLi* | Flanking markers | Site (cM) | *QTLj* | Flanking markers | Site (cM) | Environ. | *aa* | *R*^2^(*aa*)% | *aae* | *R*^2^(*aae*)% |
| --- | --- | --- | --- | --- | --- | --- | --- | --- | --- | --- | --- |
| WSCf | *Qwscf.acs-1A.1* | Xgwm633-Xgwm164 | 39.5 | *Qwscf.acs-6B.2* | Xgwm626-Xbarc24 | 96.8 | E3 | 2.94^***^ | 2.21 | 3.76^**^ | 7.20 |
|  | *Qwscf.acs-1A.2* | Xgwm497-Xpsp3003 | 109.9 | *Qwscf.acs-2B.3* | Xpsp3034-Xbarc1155 | 63.5 | E1 | 4.37^***^ | 4.35 |  |  |
|  | *Qwscf.acs-1B.2* | Xbarc61-Xwmc134 | 66.6 | *Qwscf.acs-3B.5* | Xgwm285-Xbarc1077 | 65.9 | E1 | 5.51^***^ | 6.94 |  |  |
|  | *Qwscf.acs-2A* | Xmag1730-Xwmc794 | 78.5 | *Qwscf.acs-7A.3* | Xgwm471-Xgwm60 | 1.9 | E3 | 3.54^***^ | 3.21 | 3.56^***^ | 6.48 |
|  | *Qwscf.acs-2B.4* | Xbarc159-Xksum45 | 110.6 | *Qwscf.acs-6D.1* | Xwmc113-Xpsp3200 | 14.0 | E1 | 2.79^***^ | 1.78 | 3.86^**^ | 6.81 |
|  | *Qwscf.acs-3A* | Xbarc193-Xgwm155 | 118.2 | *Qwscf.acs-7D.2* | Xgwm473-Xwmc488 | 32.1 | E3 | -3.90^***^ | 3.89 |  |  |
|  | *Qwscf.acs-3B.4* | Xbarc173-Xgwm284 | 43.6 | *Qwscf.acs-5A.2* | Xbarc165-Xwmc705 | 15.8 | E2 | 4.43^***^ | 7.56 |  |  |
|  | *Qwscf.acs-6B.1* | Xwmc341-Xbarc198 | 69.4 | *Qwscf.acs-7D.1* | Xwmc438-Xbarc172 | 25.7 | E2 | 3.23^***^ | 4.01 |  |  |
|  | *Qwscf.acs-6D.2* | Xgwm582-Xwmc469 | 31.6 | *Qwscf.acs-7A.4* | Xwmc336-Xbarc154 | 64.6 | E1 | 3.91^***^ | 3.49 |  |  |
| WSCg | *Qwscg.acs-1B.1* | Xmag2064-Xwmc694 | 45.4 | *Qwscg.acs-1B.2* | Xwmc582-Xgwm374 | 55.7 | E2 | -5.47^***^ | 3.52 | -2.62^**^ | 1.62 |
|  | *Qwscg.acs-1B.3* | Xbarc181-Xbarc61 | 61.6 | *Qwscg.acs-4B.3* | Xgwm540-Xcfd2 | 14.6 | E2 | 5.62^***^ | 3.71 |  |  |
|  | *Qwscg.acs-1B.4* | Xwmc44-Xwmc367 | 111.4 | *Qwscg.acs-4D* | Xwmc489-Xgdm61 | 40.7 | E3 | 6.55^***^ | 5.09 |  |  |
|  | *Qwscg.acs-2B.1* | Xwmc272-Xgwm630 | 15.4 | *Qwscg.acs-5B* | Xbarc116-Xksum253 | 2.0 | E3 | -4.15^***^ | 2.04 | -6.80^***^ | 10.95 |
|  | *Qwscg.acs-2B.2* | Xgdm87-Xgwm388 | 54.0 | *Qwscg.acs-7D.3* | Xgwm473-Xwmc488 | 32.1 | E2 | 3.31^***^ | 1.29 |  |  |
|  | *Qwscg.acs-2B.3* | Xmag956-Xbarc159 | 105.1 | *Qwscg.acs-3B.2* | Xgwm72-Xcfd6 | 53.6 | E2 | -4.21^***^ | 2.08 |  |  |
|  | *Qwscg.acs-2D.2* | Xcfd53-Xwmc453 | 15.0 | *Qwscg.acs-6D.1* | Xgwm582-Xwmc469 | 31.6 | E3 | 5.75^***^ | 3.91 |  |  |
|  | *Qwscg.acs-3B.1* | Xgwm284-Xpsp3112 | 48.4 | *Qwscg.acs-7A.3* | Xwmc596-Xgwm260 | 48.5 | E3 | -4.99^***^ | 2.96 | -5.23^**^ | 6.49 |
|  | *Qwscg.acs-3B.3* | Xbarc68-Xgwm285 | 64.5 | *Qwscg.acs-7D.2* | Xwmc702-Xcfd46 | 16.8 | E2 | 3.27^***^ | 1.25 |  |  |
|  | *Qwscg.acs-4A* | Xgwm165-Xmag1353 | 0.0 | *Qwscg.acs-5A.3* | Xmag781-Xgwm304 | 22.9 | E1 | -4.24^***^ | 2.55 |  |  |
|  | *Qwscg.acs-4B.4* | Xgwm6-Xksum238 | 106.8 | *Qwscg.acs-7B.2* | Xwmc517-Xbarc315 | 87.0 | E1 | -4.09^***^ | 2.36 | 6.05^***^ | 10.35 |
|  | *Qwscg.acs-5A.2* | Xwmc705-Xmag781 | 20.4 | *Qwscg.acs-7D.1* | Xbarc184-Xgwm635 | 2.0 | E2 | -3.48^***^ | 1.43 |  |  |
|  | *Qwscg.acs-5D* | Xbarc110-Xwmc161 | 61.7 | *Qwscg.acs-7B.2* | Xwmc517-Xbarc315 | 87.0 | E1 | -6.49^***^ | 5.96 |  |  |
|  | *Qwscg.acs-6D.1* | Xgwm582-Xwmc469 | 31.6 | *Qwscg.acs-7A.5* | Xwmc9-Xbarc108 | 85.9 | E1 | 6.92^***^ | 6.76 |  |  |
|  |  |  |  |  |  |  | E3 | 5.85^***^ | 4.05 | 6.94^***^ | 11.42 |
|  | *Qwscg.acs-7A.2* | Xmag3023-Xwmc596 | 40.9 | *Qwscg.acs-7A.4* | Xwmc422-Xwmc336 | 59.0 | E2 | 2.96^***^ | 1.03 |  |  |
|  | *Qwscg.acs-7A.6* | Xbarc195-Xbarc121 | 115.2 | *Qwscg.acs-7B.1* | Xbarc176-Xgwm112 | 33.7 | E3 | 5.94^***^ | 4.19 |  |  |
| WSCm | *Qwscm.acs-1A.1* | Xcfa2513-Xksum104 | 7.5 | *Qwscm.acs-4B.2* | Xksum244-Xmag2055 | 56.7 | E2 | 3.29^***^ | 2.28 |  |  |
|  | *Qwscm.acs-1A.2* | Xgwm135-Xwmc304 | 56.4 | *Qwscm.acs-5B* | Xgwm408-Xwmc75 | 99.7 | E3 | 1.65^**^ | 1.38 | -2.65^***^ | 7.08 |
|  | *Qwscm.acs-1B.2* | Xwmc134-Xcfd48 | 73.5 | *Qwscm.acs-6D* | Xwmc113-Xpsp3200 | 14.0 | E2 | -3.62^***^ | 2.76 |  |  |
|  | *Qwscm.acs-2B.2* | Xgwm132-Xcfa2278 | 41.6 | *Qwscm.acs-4B.1* | Xgwm192-Xcfd22 | 55.4 | E3 | 2.62^***^ | 3.48 | -2.25^**^ | 5.10 |
|  | *Qwscm.acs-2B.3* | Xgwm501-Xmag956 | 93.3 | *Qwscm.acs-5D* | Xwmc443-Xgwm272 | 73.0 | E2 | -2.89^***^ | 1.77 |  |  |
|  | *Qwscm.acs-2D* | Xksum44.2-Xwmc18 | 16.0 | *Qwscm.acs-4A* | Xwmc420-Xgwm601 | 12.8 | E3 | -2.30^***^ | 2.67 | 2.28^**^ | 5.22 |
| WRRpr | *Qwrrpr.acs-1A.2* | Xwmc611-Xwmc20 | 58.8 | *Qwrrpr.acs-3B.2* | Xwmc231-Xbarc173 | 43.4 | E3 | -2.59^***^ | 11.04 | -1.74^**^ | 10.05 |
|  | *Qwrrpr.acs-1A.3* | Xwmc385-Xwmc312 | 103.9 | *Qwrrpr.acs-6D* | Xwmc113-Xpsp3200 | 14.0 | E3 | -1.13^***^ | 3.68 | -1.17^***^ | 7.42 |
|  | *Qwrrpr.acs-1B.2* | Xgwm11-Xwmc626 | 38.8 | *Qwrrpr.acs-2D.2* | Xwmc601-Xksum232 | 28.0 | E2 | 1.35^***^ | 5.25 |  |  |
|  | *Qwrrpr.acs-2A.2* | Xcfd168-Xgwm312 | 130.1 | *Qwrrpr.acs-5B* | Xwmc508-Xmag959 | 125.5 | E3 | 0.90^***^ | 2.31 |  |  |
|  | *Qwrrpr.acs-2B.2* | Xmag4281-Xgwm501 | 91.7 | *Qwrrpr.acs-7A.2* | Xgwm233-Xbarc1167 | 0.0 | E3 | 0.94^***^ | 2.50 |  |  |
|  | *Qwrrpr.acs-2D.3* | Xbarc219-Xgwm349 | 95.9 | *Qwrrpr.acs-3A.1* | Xgdm43-Xwmc11 | 5.1 | E3 | -0.86^***^ | 2.36 | 1.32^***^ | 10.25 |
|  | *Qwrrpr.acs-3A.2* | Xbarc105-Xbarc1113 | 111.9 | *Qwrrpr.acs-7D* | Xwmc634-Xbarc76 | 62.7 | E2 | 1.78^***^ | 5.25 |  |  |
|  | *Qwrrpr.acs-5A.4* | Xwmc492-Xgwm96 | 58.8 | *Qwrrpr.acs-7A.3* | Xwmc603-Xwmc116 | 90.8 | E3 | 1.32^***^ | 5.74 |  |  |
| WRRps | *Qwrrps.acs-1A.2* | Xwmc24-Xpsp3151 | 26.8 | *Qwrrps.acs-6A.1* | Xwmc256-Xwmc807 | 26.7 | E1 | -1.40^***^ | 2.66 |  |  |
|  | *Qwrrps.acs-1A.2* | Xwmc24-Xpsp3151 | 26.8 | *Qwrrps.acs-1D.1* | Xwmc216-Xgwm642 | 34.2 | E3 | 1.67^***^ | 3.73 | -2.03^**^ | 11.03 |
|  | *Qwrrps.acs-1B.3* | Xmag981-Xwmc673 | 89.1 | *Qwrrps.acs-3B.4* | Xpsp3035-Xgwm299 | 112.2 | E1 | -1.64^***^ | 3.66 |  |  |
|  | *Qwrrps.acs-1D.2* | Xwmc732-Xmag3229 | 43.9 | *Qwrrps.acs-7A.3* | Xwmc336-Xbarc154 | 64.6 | E1 | -1.31^***^ | 2.32 | 1.90^**^ | 9.73 |
|  | *Qwrrps.acs-2A.2* | Xgwm30-Xgwm614 | 14.9 | *Qwrrps.acs-2D.3* | Xwmc243-Xcfd239 | 70.1 | E2 | -2.59^***^ | 5.12 | -2.14^***^ | 6.97 |
|  | *Qwrrps.acs-2A.3* | Xwmc819-Xbarc5 | 119.2 | *Qwrrps.acs-4B.1* | Xgwm495-Xgwm251 | 76.5 | E2 | -2.93^***^ | 6.54 |  |  |
|  | *Qwrrps.acs-2B.2* | Xcfa2278-Xgwm55 | 46.8 | *Qwrrps.acs-3B.3* | Xbarc173-Xgwm284 | 45.6 | E2 | 2.53^***^ | 4.88 |  |  |
|  | *Qwrrps.acs-3A* | Xwmc343-Xksum28 | 68.4 | *Qwrrps.acs-4D* | Xwmc48-Xcfd106 | 19.9 | E1 | 1.92^***^ | 4.98 |  |  |
|  | *Qwrrps.acs-4B.2* | Xwmc16-Xbarc1045 | 28.5 | *Qwrrps.acs-6A.3* | Xksum247-Xbarc113 | 98.1 | E2 | 2.73^***^ | 5.67 | 2.48^**^ | 9.34 |
|  | *Qwrrps.acs-5A.5* | Xgwm639-Xwmc492 | 54.7 | *Qwrrps.acs-6A.2* | Xwmc553-Xwmc179 | 58.9 | E3 | 1.73^***^ | 4.01 |  |  |
|  | *Qwrrps.acs-5A.6* | Xbarc1182-Xgwm595 | 135.8 | *Qwrrps.acs-7B* | Xgwm302-Xbarc258 | 57.9 | E2 | -1.75^***^ | 2.34 |  |  |
| WCRpr | *Qwcrpr.acs-1A.2* | Xcfa2513-Xksum104 | 7.5 | *Qwcrpr.acs-1B.4* | Xwmc626-Xwmc31 | 41.0 | E1 | -0.69^***^ | 1.18 |  |  |
|  | *Qwcrpr.acs-1A.3* | Xwmc120-Xpsp3027 | 86.8 | *Qwcrpr.acs-4A.1* | Xgwm165-Xmag1353 | 0.0 | E2 | -0.99^***^ | 2.06 | -1.02^***^ | 4.38 |
|  | *Qwcrpr.acs-1A.4* | Xbarc9-Xksum41 | 91.2 | *Qwcrpr.acs-7A.1* | Xbarc157-Xbarc23 | 37.0 | E3 | 0.86^***^ | 2.32 | 0.68^**^ | 2.90 |
|  | *Qwcrpr.acs-1A.5* | Xwmc59-Xwmc104 | 112. | *Qwcrpr.acs-5D* | Xbarc130-Xgwm190 | 0.0 | E3 | 0.87^***^ | 2.36 | 0.76^**^ | 3.60 |
|  | *Qwcrpr.acs-1B.2* | Xpsp3000-Xbarc131 | 3.4 | *Qwcrpr.acs-3A.1* | Xwmc11-Xgwm391 | 12.2 | E3 | -0.96^***^ | 2.90 | -0.80^***^ | 3.94 |
|  | *Qwcrpr.acs-1B.3* | Xgwm11-Xwmc626 | 38.8 | *Qwcrpr.acs-7A.2* | Xwmc596-Xgwm260 | 48.5 | E2 | 0.70^***^ | 1.04 | 0.66^**^ | 1.84 |
|  | *Qwcrpr.acs-1B.5* | Xwmc134-Xcfd48 | 73.5 | *Qwcrpr.acs-7A.3* | Xgwm282-Xmag828 | 121.0 | E1 | 0.78^***^ | 1.50 |  |  |
|  | *Qwcrpr.acs-2A.3* | Xgwm512-Xgwm30 | 2.0 | *Qwcrpr.acs-5A* | Xgwm154-Xmag694 | 5.8 | E1 | -0.63^***^ | 0.96 |  |  |
|  | *Qwcrpr.acs-2A.4* | Xmag1730-Xwmc794 | 78.5 | *Qwcrpr.acs-7D.1* | Xgwm635-Xgwm428 | 9.8 | E1 | -0.79^***^ | 1.56 | -0.78^***^ | 3.00 |
|  | *Qwcrpr.acs-2B.2* | Xwmc272-Xgwm630 | 15.4 | *Qwcrpr.acs-6B.3* | Xgwm626-Xbarc24 | 96.8 | E3 | -1.06^***^ | 3.50 | -1.00^***^ | 6.28 |
|  | *Qwcrpr.acs-2D* | Xbarc228-Xcfd50 | 71.1 | *Qwcrpr.acs-4A.3* | Xgwm610-Xgwm397 | 42.3 | E2 | 0.65^***^ | 0.90 |  |  |
|  | *Qwcrpr.acs-3A.2* | Xgwm494-Xpsp3047 | 81.4 | *Qwcrpr.acs-4B* | Xcfd39-Xwmc47 | 108.8 | E3 | 1.10^***^ | 3.78 | 0.90^***^ | 5.00 |
|  | *Qwcrpr.acs-4A.2* | Xwmc757-Xgwm610 | 32.9 | *Qwcrpr.acs-7B.3* | Xwmc276-Xbarc182 | 128.6 | E2 | 0.90^***^ | 1.68 | 0.80^**^ | 2.66 |
|  | *Qwcrpr.acs-4A.4* | Xcfd257-Xksum51 | 70.4 | *Qwcrpr.acs-7B.2* | Xbarc32-Xpsp3033 | 50.3 | E1 | 0.69^***^ | 1.18 | 0.62^**^ | 1.88 |
|  | *Qwcrpr.acs-4D* | Xmag1163-Xwmc399 | 81.8 | *Qwcrpr.acs-7D.2* | Xgdm67-Xmag892 | 51.3 | E1 | -0.98^***^ | 2.38 | -0.77^***^ | 2.90 |
|  | *Qwcrpr.acs-6A.2* | Xbarc1055-Xksum93 | 5.2 | *Qwcrpr.acs-7B.1* | Xgwm569-Xbarc1073 | 2.0 | E3 | -0.95^***^ | 2.80 | -0.75^**^ | 3.52 |
|  | *Qwcrpr.acs-6A.3* | Xwmc398-Xbarc190 | 12.2 | *Qwcrpr.acs-6B.1* | Xcfd13-Xwmc737 | 5.8 | E3 | -0.80^***^ | 2.00 | -0.73^**^ | 3.34 |
|  | *Qwcrpr.acs-6B.2* | Xgwm626-Xbarc24 | 94.8 | *Qwcrpr.acs-6D* | Xpsp3200-Xgwm582 | 16.6 | E2 | -1.02^***^ | 2.20 | -0.91^***^ | 3.50 |
| WCRps | *Qwcrps.acs-1A.1* | Xwmc24-Xpsp3151 | 26.8 | *Qwcrps.acs-6A.3* | Xksum93-Xwmc398 | 8.2 | E1 | -0.99^***^ | 4.15 | 0.82^***^ | 5.73 |
|  | *Qwcrps.acs-1A.2* | Xpsp3151-Xwmc93 | 29.8 | *Qwcrps.acs-3B.3* | Xpsp3035-Xgwm299 | 112.2 | E1 | 0.98^***^ | 4.08 |  |  |
|  | *Qwcrps.acs-1B* | Xwmc31-Xmag2064 | 42.5 | *Qwcrps.acs-6B.3* | Xpsp3131-Xgwm219 | 124.6 | E2 | -0.74^***^ | 2.13 | -0.75^**^ | 4.37 |
|  | *Qwcrps.acs-2A.2* | Xpsp3088-Xgwm372 | 108.8 | *Qwcrps.acs-2D.2* | Xwmc41-Xcfd62 | 49.3 | E1 | -1.08^***^ | 4.88 |  |  |
|  | *Qwcrps.acs-2A.3* | Xwmc819-Xbarc5 | 119.2 | *Qwcrps.acs-3B.1* | Xbarc173-Xgwm284 | 45.6 | E2 | -0.83^***^ | 2.67 |  |  |
|  | *Qwcrps.acs-2D.3* | Xcfd239-Xbarc228 | 70.1 | *Qwcrps.acs-6B.2* | Xgwm361-Xgwm273 | 49.1 | E2 | -0.72^***^ | 2.03 | -0.74^**^ | 4.29 |
|  | *Qwcrps.acs-3A* | Xcfd193-Xcfa2234 | 36.0 | *Qwcrps.acs-7B* | Xwmc232-Xbarc94 | 146.1 | E2 | 0.66^***^ | 1.71 |  |  |
|  | *Qwcrps.acs-3B.2* | Xwmc236-Xmag3356 | 92.0 | *Qwcrps.acs-5D.1* | Xwmc115-Xgwm174 | 22.6 | E1 | -0.85^***^ | 3.05 | -0.87^***^ | 6.41 |
|  | *Qwcrps.acs-5A.3* | Xwmc492-Xgwm96 | 58.8 | *Qwcrps.acs-7D* | Xbarc184-Xgwm635 | 2.0 | E2 | -0.77^***^ | 2.31 |  |  |
|  | *Qwcrps.acs-5B.1* | Xbarc59-Xbarc232 | 62.4 | *Qwcrps.acs-7A.5* | Xpsp3050-Xbarc1034 | 25.7 | E3 | 1.07^***^ | 4.67 |  |  |
|  | *Qwcrps.acs-5B.2* | Xwmc740-Xbarc308 | 134.0 | *Qwcrps.acs-5D.2* | Xcfd156-Xcfd183 | 36.7 | E2 | -0.72^***^ | 2.05 |  |  |
| GWMS | *Qgwms.acs-1A.2* | Xcfa2513-Xksum104 | 7.5 | *Qgwms.acs-4B.3* | Xgwm6-Xksum238 | 106.8 | E2 | 0.03^***^ | 5.80 |  |  |
|  | *Qgwms.acs-1A.3* | Xwmc254-Xwmc385 | 100.9 | *Qgwms.acs-4B.2* | Xgwm192-Xcfd22 | 55.4 | E2 | -0.04^***^ | 8.10 |  |  |
|  | *Qgwms.acs-1B* | Xpsp3000-Xbarc131 | 3.4 | *Qgwms.acs-3A.1* | Xwmc11-Xgwm391 | 12.2 | E1 | 0.04^***^ | 2.78 | 0.03^***^ | 5.26 |
|  | *Qgwms.acs-1B* | Xpsp3000-Xbarc131 | 3.4 | *Qgwms.acs-3A.2* | Xgwm391-Xmag4305 | 12.9 | E3 | -0.04^***^ | 3.62 | -0.04^***^ | 6.54 |
|  | *Qgwms.acs-3B.1* | Xpsp3144-Xwmc236 | 86.5 | *Qgwms.acs-5A.4* | Xcfa2185-Xbarc230 | 131.0 | E1 | -0.04^***^ | 3.12 | -0.04^***^ | 6.24 |
|  | *Qgwms.acs-3B.2* | Xwmc236-Xmag3356 | 92.0 | *Qgwms.acs-5A.4* | Xcfa2185-Xbarc230 | 131.0 | E3 | 0.03^***^ | 2.50 | 0.03^**^ | 5.36 |
|  | *Qgwms.acs-3D* | Xgdm645-Xwmc529 | 59.9 | *Qgwms.acs-6B* | Xmag3469-Xgwm644 | 17.7 | E1 | -0.03^***^ | 2.64 | -0.04^***^ | 7.66 |
|  |  |  |  |  |  |  | E3 | -0.04^***^ | 3.10 | -0.04^***^ | 8.32 |
|  | *Qgwms.acs-5D* | Xbarc110-Xwmc161 | 61.7 | *Qgwms.acs-6D.2* | Xgwm582-Xwmc469 | 31.6 | E2 | 0.04^***^ | 8.96 |  |  |
|  | *Qgwms.acs-6D.1* | Xwmc113-Xpsp3200 | 4.0 | *Qgwms.acs-7A.2* | Xcfd2049-Xwmc405 | 21.0 | E1 | -0.03^***^ | 2.48 | -0.04^***^ | 5.90 |
|  |  |  |  |  |  |  | E3 | -0.04^***^ | 2.78 | -0.04^**^ | 5.26 |

WSC, water-soluble carbohydrate concentration; WSCf, WSC at the anthesis stage; WSCg, WSC at the grain-filling stage; WSCg, WSC at the grain-filling stage; WSCm, WSC at the maturity stage; WRRpr, pre-anthesis WSC remobilization rate; WRRps, post-anthesis WSC remobilization rate; WCRpr, pre-anthesis WSC contribution rate; WCRps, post-anthesis WSC contribution rate; GWMS, grain weight of main spike. Site (cM), the most likely position of the putative QTL on the specific chromosome. E1 to E3 represent field trials at Anning farm station, Gansu, China (103º51'E, 36º04'N, 1600 m ASL) in 2012-2013, at Yongdeng farm station, Gansu, China (103º18'E, 36º42'N, 1950 m ASL) in 2013-2014, and at Yuzhong farm station, Gansu, China (104º07'E, 35º51'N, 1900 m ASL) in 2014-2015, respectively. QTL*_i_* and QTL*_j_* are a pair of QTL detected by two–dimensional searching. *aa*, the direction of the epistatic effect, of which a positive value means that the parent-type effect is greater than the recombinant-type effect and a negative value means that the parent-type effect is less than the recombinant-type effect. *R*^2^(*aa*)(%), the phenotypic variance explained by epistatic QTL. *aae*, the epistatic QTL×environment interaction effect, of which a positive value indicates *aae* effect having an increasing effect on the trait value in DS conditions and a negative value means *aae* effect having a decreasing effect on the trait value in WW conditions. *R*^2^(*aae*)(%), the phenotypic variance explained by epistatic QTL×environment interaction. ^**^*P*＜0.005, ^***^*P*＜0.001.

**Table S5 The specific dates of major growth stages recorded in the wheat RIL population under drought-stressed (DS) and well-watered (WW) conditions in different environments**

| Environ. | Water condition | Heading date  (Zadoks 55) | Anthesis date  (Zadoks 60) | grain filling  (Zadoks 71) | Maturity date  (Zadoks 92) |
| --- | --- | --- | --- | --- | --- |
| E1 | DS | Apr. 19-24, 2013 | May 3-5, 2013 | May 19-21, 2013 | Jun. 29-Jul. 1, 2013 |
|  | WW | Apr. 26-29, 2013 | May 10-13, 2013 | May 26-29, 2013 | Jul. 5-7, 2013 |
| E2 | DS | Apr. 25-28, 2014 | May 9-11, 2014 | May 25-28, 2014 | Jul. 7-9, 2014 |
|  | WW | May 2- 5, 2014 | May 17-19, 2014 | May 31-Jun.2, 2014 | Jul. 15-17, 2014 |
| E3 | DS | Apr. 23-27, 2015 | May 6-8, 2015 | May 21-23, 2015 | Jul. 4-6, 2015 |
|  | WW | Apr. 29-May 2, 2015 | May 12-15, 2015 | May 28-30, 2015 | Jul. 11-13, 2015 |

E1 to E3 represent field trials at Anning farm station, Gansu, China (103º51'E, 36º04'N, 1600 m ASL), at Yongdeng farm station, Gansu, China (103º18'E, 36º42'N, 1950 m ASL), and at Yuzhong farm station, Gansu, China (104º07'E, 35º51'N, 1900 m ASL), respectively. Herein growth stages were referred to decimal codes described by Zadoks et al. [58].
